# Dual functions of silibinin in attenuating aortic dissection via regulating iron homeostasis and endoplasmic reticulum stress against ferroptosis

**DOI:** 10.1038/s41419-024-07309-x

**Published:** 2024-12-18

**Authors:** Zhen Qi, Qiu-Guo Wang, Meng-Xi Huang, Yi-Fan Zeng, Jing-Yu Li, Zhi-Cheng Duan, Ling Tan, Hao Tang

**Affiliations:** 1 Department of Cardiovascular Surgery, the Second Xiangya Hospital, Central South University, Changsha, China; 2 Jinshan Hospital, Fudan University, Shanghai, China

**Keywords:** Aortic diseases, Cell death

## Abstract

Aortic dissection (AD) poses a significant threat to cardiovascular health globally, yet its underlying mechanisms remain elusive. Smooth muscle cells death and phenotypic switching are critically important pathological processes in AD. Currently, no pharmacological therapies have proven effective in managing AD. This study aims to elucidate the involvement of ferroptosis in AD progression and explore ferroptosis inhibition as a potential therapeutic approach for AD management. Elevated expression of ferroptosis markers (HMOX1, ACSL4, and 4-HNE) was observed in AD patients and β-Aminopropionitrile (BAPN)-induced mice. In vivo administration of silibinin (SIL) attenuated aortic dilation, inflammation, mitochondrial injury, and ferroptosis. SIL treatment enhanced cell viability and mitochondrial function while reducing reactive oxygen species (ROS) generation and mitigating ferroptosis in primary human aortic smooth muscle cells (HASMCs) induced by RSL3 or IKE. Mechanistically, RNA-sequencing analysis identified dysregulation of iron homeostasis and endoplasmic reticulum stress, which were modulated by SIL. Molecular docking, cellular thermal shift assay, drug affinity responsive target stability, and surface plasmon resonance analysis confirmed HMOX1 as a direct target of SIL, highlighting its role in modulating iron homeostasis. Moreover, NCT-502, a PHGDH inhibitor, reversed the protective effect of SIL in RSL3-induced HASMCs. Conversely, 4-PBA and ZnPP demonstrate a facilitative role. This suggests that SIL plays a crucial role in ferroptosis development by modulating iron homeostasis and endoplasmic reticulum stress-mediated serine biosynthesis, both in vitro and in vivo. Iron homeostasis and endoplasmic reticulum stress of HASMCs drive the development of aortic dissection. These findings unveil a novel role of SIL in mitigating ferroptosis in HASMCs, offering a promising therapeutic avenue for treating AD.

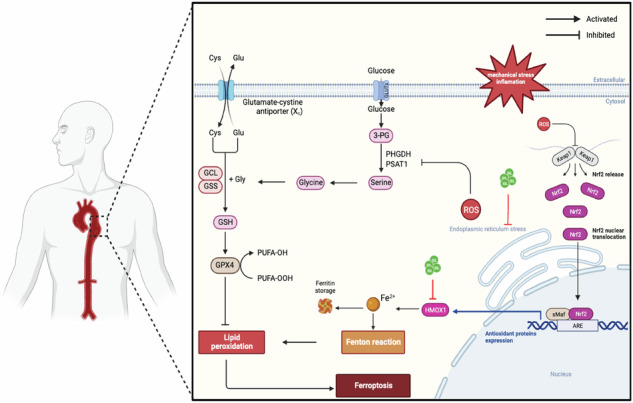

## Introduction

Aortic dissection (AD) is a severe cardiovascular emergency, occurring at an estimated annual incidence of approximately 3–6 cases per 100,000 individuals. It results in rapid hemodynamic decline, characterized by severe circulatory failure due to cardiac tamponade, aortic rupture, and multi-organ failure stemming from inadequate perfusion [1, 2].

AD is primarily caused by degeneration or cystic necrosis of the aortic media, leading to blood leakage through an intimal tear and the formation of a false lumen between the intima and the media [3]. Without surgical intervention, patients with AD face a mortality rate of 1%-2% per hour post-onset, with up to 90% mortality within 90 days [4]. Currently, open surgery remains the most effective treatment for AD [5].

Smooth muscle cells (SMCs) in the medial layer of the aorta are essential for maintaining the vascular architecture and stability of the vessel wall under the stress of blood flow. SMCs death and extracellular matrix remodeling are the major causes of pathological changes in the aortic wall and are the pathological basis for AD development [6, 7]. In recent years, several types of programmed cell death, such as apoptosis, pyroptosis, and necroptosis, have been reported to be involved in vascular smooth muscle death in AD [6, 8]. Studies have shown that inhibitors of IRE1α kinase reduce vascular smooth muscle cell apoptosis, thereby attenuating AD [9]. Melatonin prevents thoracic aortic aneurysm and dissection through Sirt1-dependent regulation of oxidative stress and vascular smooth muscle cell apoptosis [10]. Despite efforts utilizing specific gene editing techniques and drug targeting of apoptosis or pyroptosis, complete reversal of Ang II-induced aortic smooth muscle cell death has not been achieved. Currently, the specific molecular mechanisms of aortic smooth muscle cell death are unknown, and there is a lack of effective clinical medical therapy to intervene in the development of AD. More intensive methods have been employed to enhance understanding of AD, but they have independently contributed to adverse outcomes. Despite these efforts, these strategies often prove inadequate, and the mechanisms underlying AD remain elusive.

Ferroptosis is characterized by lipid peroxidation, mitochondrial damage, and accumulation of free iron. It is also accompanied by alterations in a specific set of genes (ACSL4, GPX4, HMOX1). Recent studies have demonstrated that ferroptosis plays a vital role in the pathophysiological processes involved in a variety of cardiovascular diseases, including cardiomyopathy [11, 12], myocardial infarction [13], and heart failure [14, 15]. However, research on ferroptosis in AD is limited. Recent studies have indicated that ferroptosis contributes to the pathological progression of AD and targeting it may offer a novel therapeutic approach for AD treatment [7]. Another study has suggested that ferroptosis may result in the reduction of SMCs, exacerbating the pathological process of AD [16]. The large accumulation of lipid peroxides is considered a key feature of ferroptosis [17]. It was found that superoxide dismutase, which inhibits the production of reactive oxygen species (ROS), was significantly downregulated in the middle layer of the aorta of patients with thoracic aortic dissection compared to the normal aorta. Additionally, lipid peroxidation levels were markedly increased in the aortic tissues of patients with AD [18].

Silibinin (SIL), an active ingredient extracted from the seeds of *Silybum marianum*, is known for its hepatoprotective properties and is often referred to as a “natural hepatoprotective drug”. It has been used in herbal medicine for thousands of years and has been approved for widespread clinical use due to numerous clinical studies [19–22]. Several studies have demonstrated the potential protective and therapeutic applications of SIL in cardiovascular diseases (CVDs), particularly in terms of its antioxidant, hypo-lipidemic, hypo-glycaemic, anti-hypertensive, and cardioprotective effects [23–27]. A recent study has shown that SIL can protect HepG2 cells against ferroptosis by targeting ACSL4 to inhibit lipid peroxidation [28]. However, it is unclear whether SIL can modulate SMCs ferroptosis in AD. The precise mechanism by which SIL influences the onset and progression of AD remains incompletely understood.

To our knowledge, the impact and mechanism of SIL on ferroptosis have not been previously investigated in the context of AD. Here, we elucidate the pivotal role of SIL in mitigating ferroptosis by directly modulating HMOX1 to regulate iron homeostasis and inhibiting endoplasmic reticulum stress (ERS) to enhance serine metabolism. This study provides insight into SIL as a potential therapeutic approach for AD by targeting ferroptosis to confer protective effects on SMCs.

## Materials and methods

### Human aortic samples

Human aortic samples were obtained from patients undergoing open surgical repair. Healthy control samples were collected from brain death patients or organ donors who did not have aortic diseases. All procedures were conducted in accordance with the principles outlined in the Declaration of Helsinki. Informed consent for aortic specimen collection was obtained from each study participant and approved by the Medical Ethical Committee of the Second Xiangya Hospital, Central South University, Changsha, China (Ethics Approval No. LYF20230192). Baseline demographic information, biochemical, and hematological data, risk factors, and clinical outcomes were collected and are listed in Supplementary Table S1.

### Animal models

The Animal Care and Use Committee of the Second Xiangya Hospital, Central South University approved all animal experiments in this study (Ethics Approval No. 20240291). Three-week-old male C57BL/6J mice, obtained from Beijing Vital River Laboratory Animal Technology Co., Ltd, were maintained under standard conditions (temperature: 24 ± 2 °C, humidity: 50–60%, 12-h light/dark cycle) with unlimited access to food and water. To induce aortic dissection, the mice were given drinking water containing 0.3% β-aminopropionitrile (BAPN, A3134, Sigma-Aldrich) for 4 weeks, followed by a 24-h infusion of angiotensin II (AngII, 1000 ng/kg/min) using Alzet osmotic pumps (1003D).

### Cell culture

Human aortic smooth muscle cells (HASMCs) were obtained from ScienCell Research Laboratories and cultured in smooth muscle cell medium (ScienCell, United States) supplemented with 2% fetal bovine serum (FBS), 1% Smooth Muscle Cell Growth Supplement, and 1% penicillin/streptomycin (P/S) solution at 37 °C and an incubator temperature of 5% CO_2_. HEK293T cells were purchased from the ATCC and cultured in DMEM (Gibco, United States) supplemented with 10% FBS and 1% P/S solution at 37 °C in a 5% CO_2_ incubator. Upon reaching 70–80% confluence, cells were detached using 0.05% trypsin and reseeded for subsequent experiments. HASMCs in passages 3–6 were utilized in all experiments. Following medium refreshment, HASMCs were treated with IKE (2.5 μM) for 18 h or RSL3 (50 nM) for 12 h to induce ferroptosis, after which the cells were harvested for further analysis.

### Lentivirus vector-mediated NFE2L2 overexpression in HASMCs

The NFE2L2 open reading frame (ORF) sequence was synthesized and inserted into a GV658 vector by GeneChem (Shanghai, China). The Lenti-X 293T cell line was utilized to produce lentivirus, with the assistance of packaging plasmids (psPAX2, pMD2.G). In brief, HASMCs were cultured in T25 flasks until reaching 70–80% confluence, followed by lentiviral infection (1.0 × 10^9^ TU/mL) for 16 h at a multiplicity of infection (MOI) of 50. HASMCs were then infected with lentivirus overexpressing NFE2L2 (OE-NRF2) or control lentivirus (OE-vector) for subsequent experiments.

### Cell transfection

The siRNA targeting HMOX1 was designed and synthesized by Bioegene (Shanghai, China). Transfection of HASMCs was performed using Lipofectamine 2000 (Invitrogen). The HASMCs were reseeded into 6-cm dishes one day prior to transfection. Once they reached 70-80% confluency, the cells were transfected with either a negative control (NC) or siRNA-HMOX1 at a concentration of 30 pmol. The detailed sequences are provided in Supplementary Table S2.

### Ultrasonographical assessment of the aorta

Aortic diameter was evaluated using a Mindray M9 ultrasound machine (Mindray, USA). Mice were anesthetized with 1.5% isoflurane, and after carefully removing the fur from the left chest and abdomen, an ultrasound was performed to assess heart rate and aortic diameter.

### Transmission electron microscopy

HASMCs and fresh aortic tissue samples were collected and fixed overnight at 4 °C in a 2.5% glutaraldehyde solution. Following dehydration, permeation, and embedding, ultrathin sections were prepared. Mitochondrial size, membrane structure, and crest morphology were analyzed using a Hitachi HT-7800 transmission electron microscope.

### RNA-sequencing and bioinformatics analysis

For RNA-sequencing of HASMCs, three experimental groups were established: the control group, the RSL3 group, and the RSL3 with Silibinin treatment (RSL3 + SIL) group. Total RNA was extracted and cDNA libraries were constructed. RNA sequencing was conducted by the Beijing Genomics Institute (BGI, Shenzhen) using the DNBSEQ platform. Following quantitative processing, the DEseq2 R package (version 1.42.1) was employed for differential analysis based on the negative binomial distribution model. DEGs were identified using a cutoff of |log2(Fold change)| ≥ 0.5 and adjusted *p*-value < 0.05.

For RNA-sequencing of human aortic tissue, aortas were obtained from both healthy donors and patients with acute aortic dissection. Total RNA was extracted and cDNA libraries were prepared. RNA sequencing was performed using the Illumina NovaSeq 6000 platform. Analysis of the sequencing data, including Heatmap analysis, gene ontology (GO) analysis, and Gene Set Enrichment Analysis (GSEA), was conducted using the Pheatmap R package (version 1.0.12), clusterProfiler R package (version 4.10.1), and Pathview R package (version 1.42.0), respectively. The raw data have been uploaded to the National Center for Biotechnology Information (NCBI) BioProjects database, with the following accession numbers: PRJNA1097277 and PRJNA1097294.

### Flow cytometry

Lipid peroxidation was assessed using C11-BODIPY 581/591 (Thermo Fisher Scientific). Briefly, HASMCs were seeded in 6-well plates and treated as indicated. C11-BODIPY 581/591 was then added to the cell culture medium supernatant at a final concentration of 2.5 μM. Following a 30-minute incubation at 37 °C in the dark and two washes with cold PBS, fluorescence microscopy was employed to visualize the HASMCs. For sample collection, HASMCs were rinsed with cold PBS and detached using 0.05% Trypsin. All samples were subsequently filtered by 70 μm cell strainers (Falcon, United States) and analyzed using the BD Biosciences FACSVerseTM flow cytometer. FlowJo 10.4 software was utilized for result analysis.

### Molecular docking

Molecular docking was performed using the Schrödinger Maestro 13.5. The X-ray crystal structure of human HMOX1 (PDB ID: 1T5P) was downloaded from the PDB database and processed using the Protein Preparation Workflow module of Schrödinger. The 3D structure of Silibinin (PubChem CID:31553) was optimized for energy using the LigPrep module of Schrödinger. The optimized compounds underwent Ligand docking using Glide in Extra Precision (XP) mode. A higher absolute value of the predicted docking score indicates a stronger binding affinity. PyMol 2.6 was used to create the 2D and 3D docking modes.

### Iron staining

The FerroOrange Assay Kit (Dojingo, China) was used to measure the intracellular ferrous ion (Fe^2+^) of HASMCs. In brief, HASMCs were washed three times with HBSS. The 1 μM FerroOrange working solution was added to the cells and incubated at 37 °C for 30 min. The HASMCs were observed by fluorescence microscopy.

### Statistical analysis

The normality of the data was evaluated using the Shapiro–Wilk (S–W) test for measurement data. The normally distributed data were expressed as mean ± standard deviation (SD). Comparisons between two groups were evaluated using Student’s t-test, while multiple group comparisons were analyzed using One-way or Two-way analysis of variance (ANOVA) followed by Tukey’s posthoc test. Statistical analyses were conducted using GraphPad Prism 10 Software. Non-normally distributed data were expressed as the median (The interquartile range (IQR)). Comparisons between the two groups were evaluated using the Mann-Whitney U test. The Chi-squared test was used to compare categorical variables between the two groups. Statistical analyses were conducted using IBM SPSS Statistics 29.0. *p* < 0.05 was considered statistically significant.

An *Expanded Methods section* is available in the Supplemental Material, which includes detailed methods on the following: Reagent, Histological staining, CCK8 Assay, Reactive oxygen species assays, Measurement of the mitochondrial membrane potential, Western blotting, RT-qPCR, Immunofluorescence staining, Ferrous iron assay, Malondialdehyde assay, Cellular thermal shift assay, Surface plasmon resonance assay, and Drug affinity responsive target stability assay.

## Results

### Ferroptosis likely occurs in the aortic of AD

**Fig. 1:**
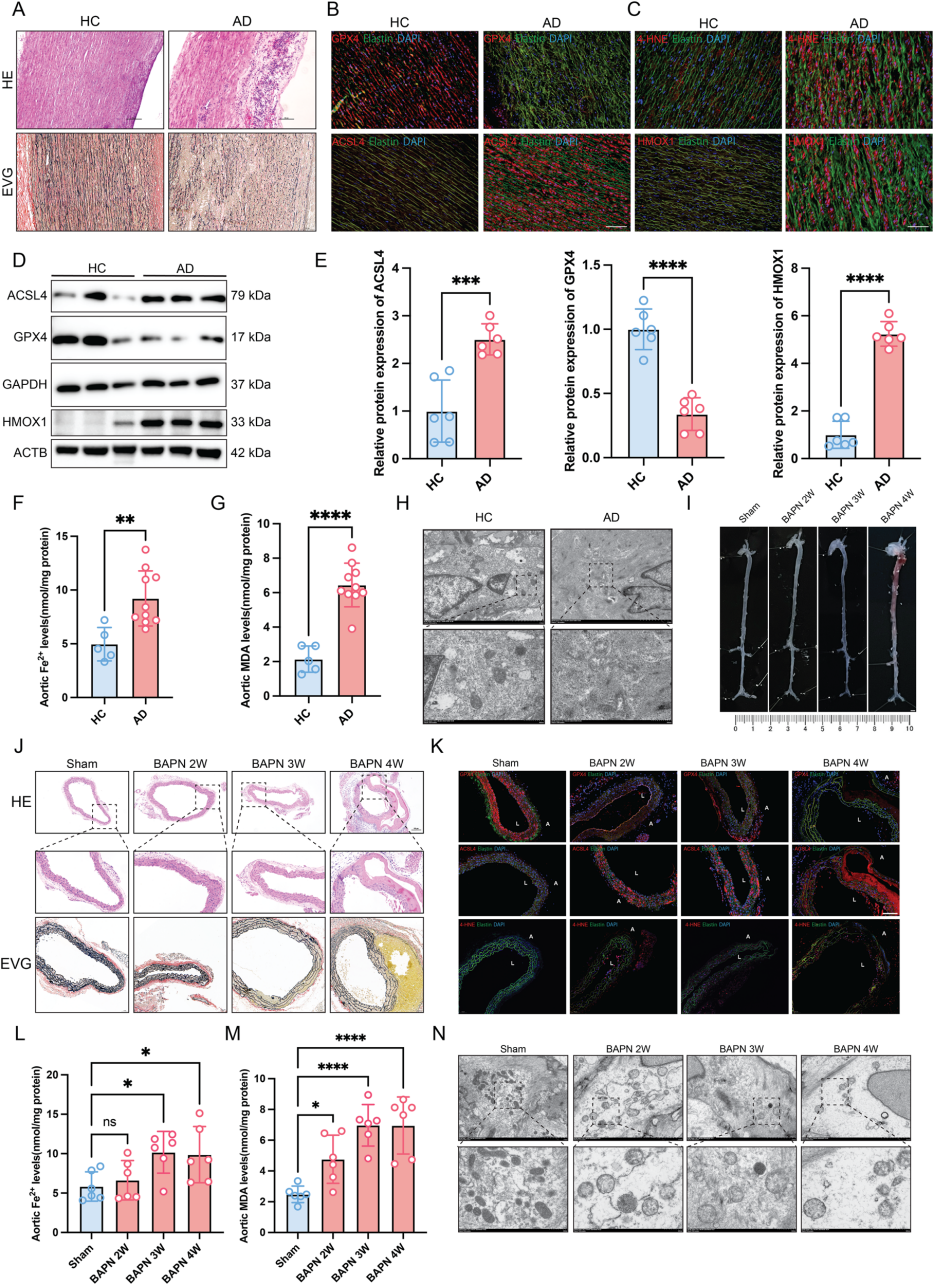
Ferroptosis occurs in the aortic tissue of AD patients and BAPN-induced AD mouse model. **A** H&E staining and EVG staining of aorta tissue from healthy control (HC) and patient (AD). **B**, **C** Representative immunofluorescence staining for GPX4, ACSL4, and 4-HNE in aorta tissue from HC and AD. Scale bar, 50 μm. **D** Immunoblots analysis for GPX4, ACSL4, and HMOX1 in aorta tissue from HC and AD. **E** Relative quantitative analysis of gray value, *n* = 6. **F** Ferrous iron levels in aortic samples from HC (*n* = 5) and AD (*n* = 10). **G** The MDA levels of human aorta from HC (*n* = 5) and AD (*n* = 10). **H** Transmission electron microscopy analysis of the mitochondria of human aorta from HC and AD. Scale bar = 500 nm. **I** The morphology of the aorta from BAPN-induced mice. Scale bar = 2 mm. **J** H&E staining and EVG staining of aorta tissue from BAPN-induced mice. **K** Representative immunofluorescence staining for GPX4, ACSL4, and 4-HNE in aorta tissue from BAPN-induced mice. Scale bar = 50 μm. **L** Ferrous iron levels in mouse aortic samples, *n* = 6. **M** The MDA levels of mouse aorta, *n* = 6. **N** Transmission electron microscopy analysis of the mitochondria of mouse aorta. Scale bar = 500 nm. Data are presented as mean ± SD. *p < 0.05, **p < 0.01, ***p < 0.001, ****p < 0.0001. ns no significant difference. A indicates adventitia; L indicates lumen.

The pathology of aortic dissection is characterized by the reduction of smooth muscle cells, infiltration of the inflammatory cells, and disruption of the elastin. Pathological staining results showed a substantial infiltration of inflammatory cells in the media of the AD patient aorta, accompanied by significant disruption and degradation of elastin (Fig. 1A). To identify whether ferroptosis plays a vital role in the pathology of AD, immunofluorescence staining was used to evaluate the expression level of the ferroptosis-associated proteins (GPX4, ACSL4, HMOX1, and 4-HNE) in the aorta. The results indicated that these proteins exhibited aberrant expression patterns in the media of the aorta in AD (Fig. 1B, C). Western blot analysis further confirmed that GPX4, ACSL4, and HMOX1 were abnormally expressed in the aorta of AD (Fig. 1D, E).

The levels of ferrous iron and MDA were markedly elevated in the aorta of AD patients relative to healthy controls, indicating that excessive ferrous iron and lipid superoxide accumulation occurs in the aortic tissue of AD patients (Fig. 1F, G). Transmission electron microscopy demonstrated that aortic ultrastructural injury occurs in the SMCs of the aorta of AD patients compared to healthy controls (Fig. 1H). This injury was characterized by mitochondrial swelling, mitochondrial fission, vacuolar degeneration, and mitochondrial cristae rarefication. To further illustrate the role of ferroptosis in the initiation and progression of AD, a mouse model was established by administering BAPN for 2, 3, or 4 weeks to study ferroptosis. No significant pathology changes were observed at the early stage (2 weeks). However, at three weeks, a mild dilatation of the ascending aorta was noted. By four weeks, the ascending aorta exhibited pronounced dilatation and dissection (Fig. 1I). Pathological staining demonstrated disorder and disruption of the elastin and dissection of the aorta (Fig. 1J). Immunofluorescence analysis showed abnormal alterations in ferroptosis markers GPX4, ACSL4, and 4-HNE during the initiation and progression of AD (Fig. 1K). The levels of ferrous iron exhibited a gradual increase in the aortas of mice following the induction of BAPN (Fig. 1L). Similarly, MDA levels were markedly elevated in the aorta of BAPN-induced mice (Fig. 1M). Transmission electron microscopy revealed that aortic ultrastructural injury was evident at the early stage (2 weeks) and progressed to more severe injury at the late stage (4 weeks) (Fig. 1N). These above results indicated that ferroptosis may be involved in the initiation and progression of AD.

### Silibinin attenuated the ferroptosis in the BAPN-induced AD mice

**Fig. 2:**
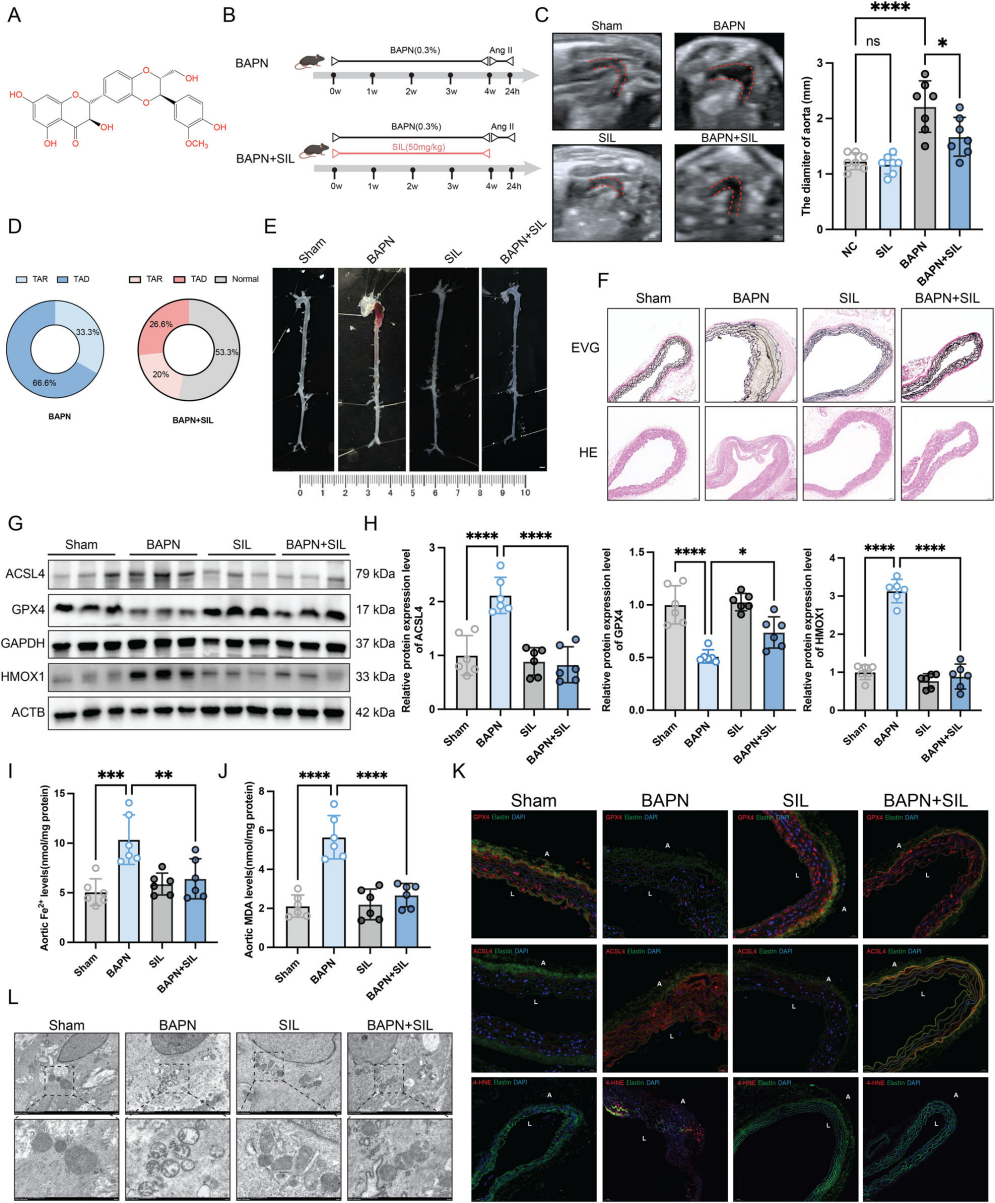
SIL attenuated the ferroptosis in the BAPN-induced AD mouse model. **A** The chemical structure of SIL. **B** Schematic diagram depicting the mouse treatment procedure. **C** Representative ultrasound images of the mouse aorta. Scale bar = 1 mm. The diameter of the aorta was measured, *n* = 7. **D** The incidence of aortic rupture and dissection. *n* = 15 per group. **E** The morphology of the aorta from BAPN-induced mice. Scale bar = 2 mm. **F** H&E staining and EVG staining of mouse aorta tissue. **G** Western blot analysis for ACSL4 and GPX4 in the mouse aorta. **H** Relative quantitative analysis of gray value, *n* = 6. **I** Ferrous iron levels in mouse aortic samples. *n* = 6. **J** The MDA levels of mouse aorta, *n* = 6. **K** Representative immunofluorescence images of the ACSL4, GPX4, and 4-HNE in mice aorta tissue. **L** Transmission electron microscopy analysis of the mitochondria of mouse aorta. Scale bar = 500 nm. Data are presented as mean ± SD. *p < 0.05, **p < 0.01, ***p < 0.001, ****p < 0.0001. ns: no significant difference. A indicates adventitia; L indicates lumen. TAD thoracic aortic dissection, TAR thoracic aortic rupture.

To illustrate the protective effect of SIL in AD, the BAPN-induced mice were intraperitoneally injected with SIL (50 mg/kg/2 days) or vehicle for 28 days, then the mice were subcutaneously infused 1000 ng/kg/min AngII for 24 h (Fig. 2B). All surviving mice underwent ultrasonography to measure the ascending aortic diameter. Ultrasound showed that there was significant dilatation of the aorta in the BAPN group, while treatment with SIL reversed the adverse effect (Fig. 2C). Notably, SIL also decreased the incidence of aortic rupture and dissection in vivo (Fig. 2D). Consistently, upon harvesting mouse aortas, we observed significant dilatation and dissection in BAPN-induced mice, which was attenuated by SIL treatment (Fig. 2E). Pathological staining demonstrated that disorder and disruption of the elastin and dissection of the aorta which induced by BAPN were significantly reversed by SIL treatment (Fig. 2F). Western blot analysis showed abnormal expression of ACSL4, GPX4, and HMOX1 in the BAPN group. SIL treatment downregulated the expression levels of HMOX1 and ACSL4 while upregulating GPX4 expression levels (Fig. 2G, H). The levels of ferrous iron and MDA were markedly elevated in the aorta of BAPN-induced mice relative to the Sham group. However, treatment with SIL resulted in a significant decrease in the accumulation of ferrous iron and MDA in the mouse aorta (Fig. 2I, J). Our immunofluorescence staining results corroborated the western blot findings, revealing aberrant expression of ACSL4, GPX4, and 4-HNE in the medial layer of the mouse aorta. Furthermore, SIL treatment notably suppressed ferroptosis in the smooth muscle cells of the mouse aorta (Fig. 2K). Mitochondrial ultrastructural damage is another hallmark of ferroptosis. Mitochondrial fission, swelling, vacuolar degeneration, and cristae rarefaction were more pronounced in the BAPN-induced mouse aorta. Importantly, SIL administration alleviated the observed mitochondrial injuries in the BAPN + SIL group (Fig. 2L). The above results suggest that SIL protects against aortic dissection as well as regulates ferroptosis.

### Silibinin alleviated ferroptosis in HASMCs

**Fig. 3:**
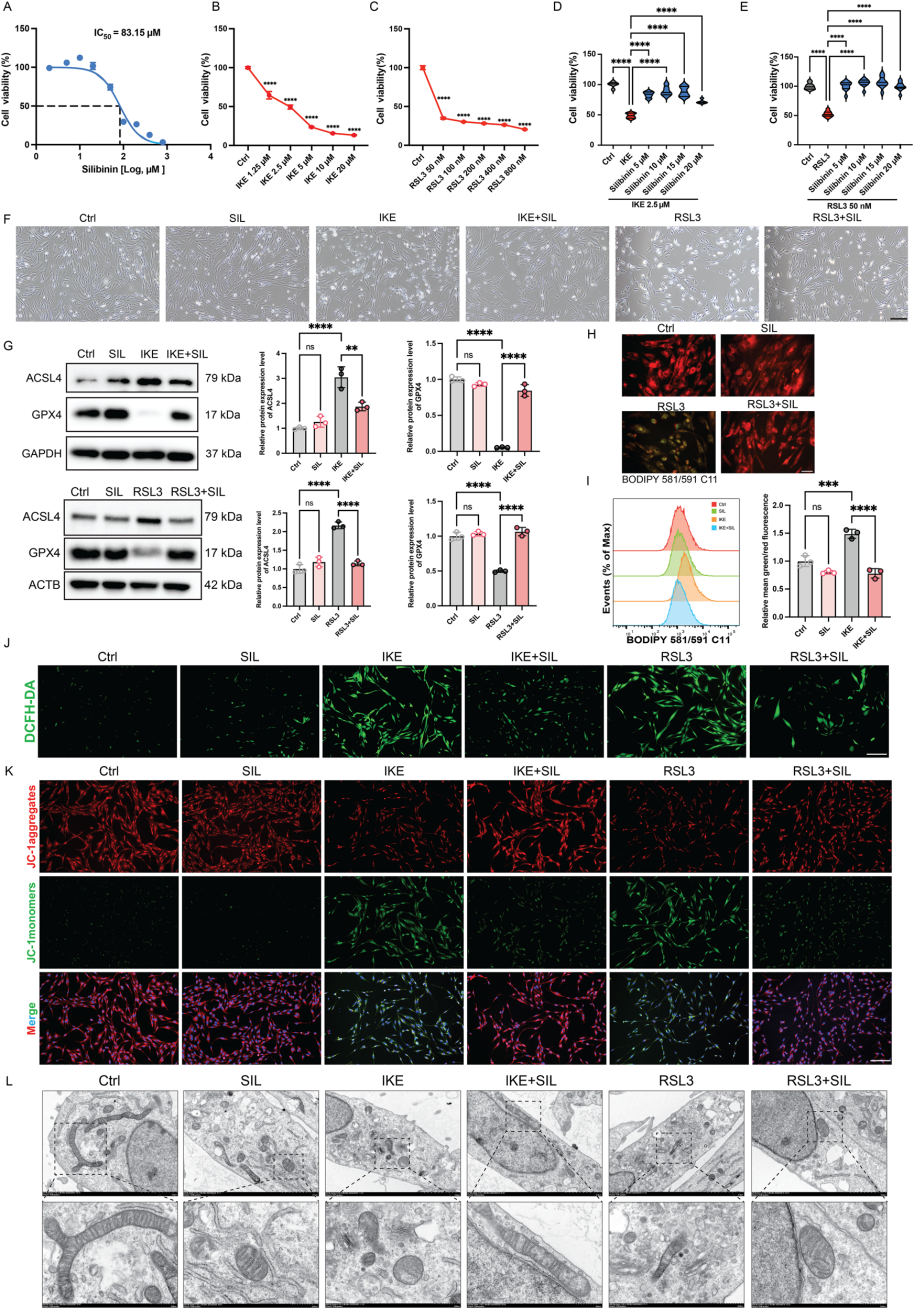
SIL inhibited ferroptosis in HASMCs. **A** The IC_50_ of SIL detected by CCK8, *n* = 10. **B** Cell viability of HASMCs treated with a gradient concentration of IKE (1.25, 2.5, 5,10, and 20 μM) for 24 h. **C** Cell viability of HASMCs treated with a gradient concentration of RSL3 (50, 100, 200, 400, and 800 nM) for 24 h. **D**, **E** Pretreatment with SIL (5, 10, 15, and 20 μM) for 24 h before IKE exposure (2.5 μM) for 18 h and RSL3 exposure (50 nM) for 12 h individually; CCK8 assay was used to assay viability of HASMCs. **F** The morphology of the HASMCs. Scale bar = 50 μm. **G** Western blot analysis for protein expression of GPX4 and ACSL4 in HASMCs. *n* = 3. **H** BODIPY 581/591 C11 probe was used to determine Lipid ROS in RSL3-induced HASMCs. Scale bar = 50 μm. **I** Flow cytometry analysis of the lipid ROS. *n* = 3. **J** DCFH-DA staining was used for the detection of ROS. Scale bar = 50 μm. **K** JC-1 staining was used to assay the mitochondria membrane potential. Scale bar = 50 μm. **L** The morphology of mitochondria in HASMCs was examined by transmission electron microscopy. Scale bar = 500 nm. Data are presented as mean ± SD. **p < 0.01, ***p < 0.001, ****p < 0.0001; ns no significant difference.

To clarify the cytotoxicity of SIL on HASMCs, we co-incubated gradient concentrations of SIL with HASMCs for 24 h. The CCK8 results showed that the IC_50_ of SIL was 83.5 μM (Fig. 3A). It showed that SIL did not affect cell viability at low concentrations (5–20 μM). Two classical inducers of ferroptosis (IKE and RSL3) were used to establish the ferroptosis cells model. Our results showed that IKE treatment (2.5 μM) reduced the viability of HASMCs to 50% (*p* < 0.0001) (Fig. 3B), while RSL3 treatment (50 nM) reduced the viability of HASMCs to 35% (*p* < 0.0001) (Fig. 3C). Subsequent experiments were conducted using these concentrations. Interestingly, pre-incubation with SIL at concentrations of 5–20 μM led to improved cell viability, with a notable enhancement observed at 10 μM against RSL3 or IKE treatment (Fig. 3D, E). Therefore, 10 μM SIL was selected for subsequent experiments. The morphological characteristics of HASMCs revealed that when treated with RSL3 or IKE, the HASMCs exhibited significant crumpling and rounding, accompanied by cell death. In contrast, both SIL and classical ferroptosis inhibitor Fer-1 demonstrated the capacity to significantly inhibit HASMCs death (Figs. 3F and S1A). Furthermore, the administration of both SIL and Fer-1 resulted in the attenuation of the aberrant expression of GPX4 and ACSL4, which were induced by the ferroptosis inducer (Figs. 3G and S1B–D). The level of reactive oxygen species (ROS) and lipid peroxidation was increased significantly in the RSL3 group and IKE group. As expected, both SIL and Fer-1 significantly inhibited the production of lipid peroxidation and ROS in vitro (Figs. 3H–J and S1E). Meanwhile, both SIL and Fer-1 reversed the adverse effect of the mitochondria (function and morphology) induced by the IKE and RSL3 in the HASMCs (Figs. 3K, L and S1F). Collectively, these findings underscore SIL’s novel role as a ferroptosis inhibitor in HASMCs.

### Oxidative stress response and serine family amino acid metabolic processes involved in HASMCs ferroptosis

**Fig. 4:**
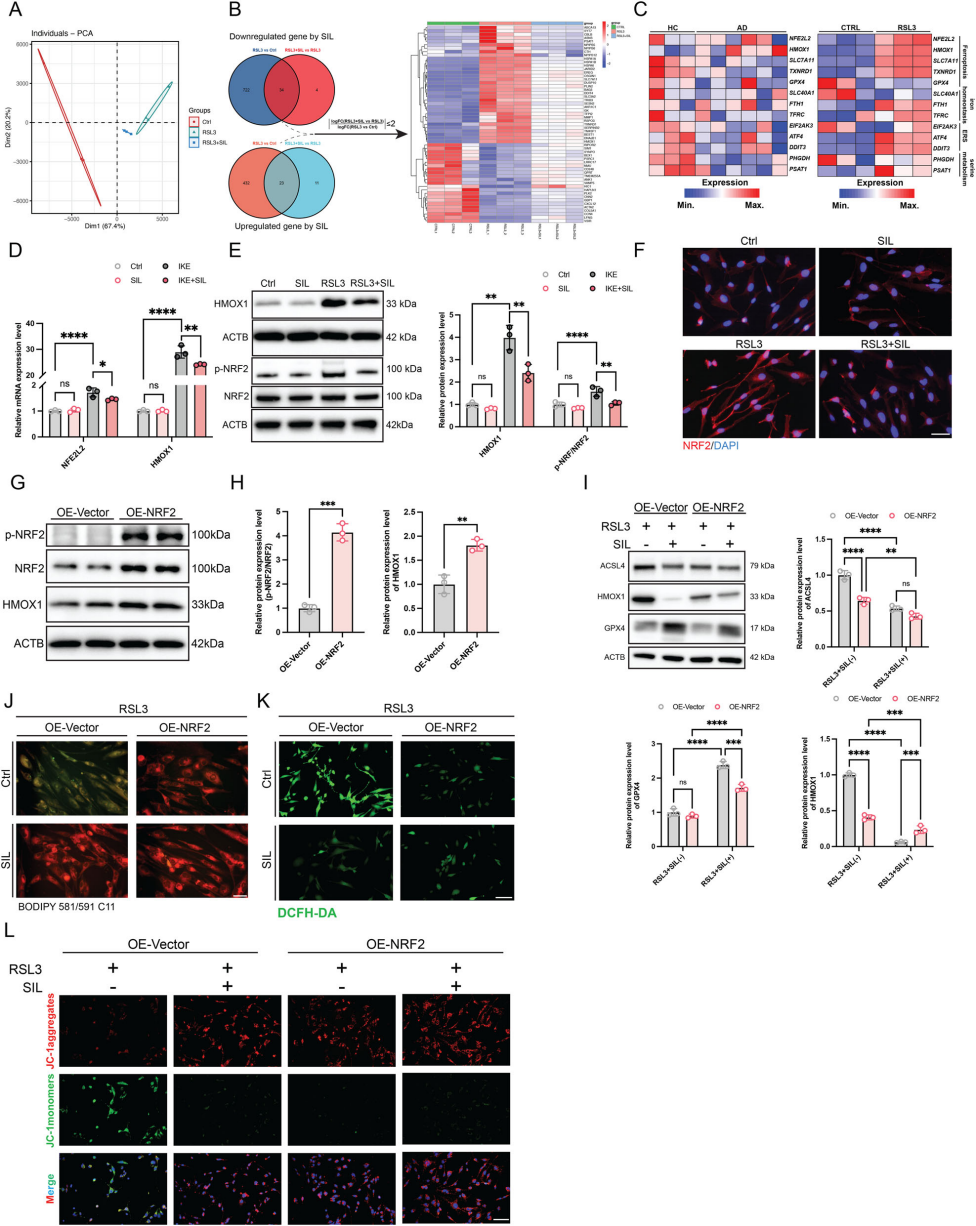
SIL rescued ferroptosis-induced dysfunction of gene expression. **A** Principal component analysis (PCA) was employed to differentiate the gene expression profiles between the control group, the RSL3 group, and the RSL3 + SIL group. **B** A Venn diagram is presented to illustrate the genes shared between two clusters, namely the RSL3 vs. Ctrl and RSL3 + SIL vs. RSL3 comparisons. The genes were found to be upregulated by RSL3 compared to the control group and then reversed by SIL treatment (upper) or downregulated by RSL3 compared to the control group and then reversed by SIL treatment (lower). A significant reversal was defined as the absolute value of the logFC (RSL3 + SIL vs. RSL3)/logFC (RSL3 vs. Ctrl) ratio being less than or equal to 2. Representative heatmap showed DEGs that were significantly altered induced by RSL3 and rescued by SIL treatment. **C** Heatmap of genes that are related to ferroptosis, iron homeostasis, and ERS in the RNA-seq analysis (Left panel: Human aorta tissue RNA-seq analysis; Right panel: RSL3 induced HASMCs RNA-seq analysis). **D** The mRNA expression levels of *NFE2L2* and *HMOX1*, *n* = 3. **E** Western blot results of p-NRF2, NRF2, and HMOX1 protein expression levels, *n* = 3. **F** Representative immunofluorescence staining images of NRF2. Scale bar, 50 μm. **G**, **H** HASMCs were infected by NFE2L2 overexpressed lentivirus (OE-NRF2) or control lentivirus (OE-vector). Immunoblots analysis and densitometric quantification of the protein expression levels of p-NRF2, NRF2, and HMOX1, *n* = 3. **I**–**L** The OE-vector HASMCs and OE-NRF2 HASMCs were pretreated with SIL for 24 h, and then treated with RSL3 for 12 h. **I** Western blot analysis and densitometric quantification of the protein expression levels of ACSL4, GPX4, and HMOX1, *n* = 3. **J** Lipid ROS staining images of RSL3-induced HASMCs. Scale bar, 50 μm. **K** The images of DCFH-DA/Hoechst 33342 staining. Scale bar, 50 μm. **L** The representative JC-1 staining images. Scale bar, 50 μm. Data are presented as mean ± SD. *p < 0.05, **p < 0.01, ***p < 0.001, ****p < 0.0001; ns no significant difference.

To further elucidate the underlying protective mechanism of the SIL against ferroptosis in AD. Subsequently, HASMCs were pretreated with SIL for 24 h and then induced with RSL3 for 12 h. RNA sequencing was conducted on HASMCs in the control group, RSL3 group, and RSL3 + SIL pretreatment (RSL3 + SIL) group. Principal component analysis (PCA) depicted the gene expression profiles between the Ctrl groups, RSL3 groups, and RSL3 + SIL groups (Fig. 4A). The differentially expressed genes (DEGs) among the three groups were subjected to further analysis. The objective was to examine which genes were upregulated or downregulated in the RSL3 group and were reversed by SIL treatment (RSL3 + SIL group) (Fig. 4B). The Venn diagram was used to identify the overlapping genes between the DEGs and ferroptosis-related genes. The 10 most enriched genes were PSAT1, HMOX1, TRIB3, SLC7A11, TXNRD1, DDIT4, SESN2, SLC3A2, ASNS, and HIC1 (Fig. S2A). The protein-protein interactivity network between the DEGs was analyzed using the STRING database (Fig. S2B). Furthermore, the Cytospace software was employed to identify the hub gene, with HMOX1 emerging as the highest-scoring gene (Betweenness: 446.9) (Fig. S2C). Among these 57 DEGs, Gene Ontology (GO) analyses indicated that oxidative stress-related biological processes and serine family amino acid metabolic processes triggered by RSL3 were abrogated by SIL (Fig. S2D). Similarly, Gene Set Enrichment Analysis (GSEA) and Pathview analysis also demonstrated that protein processing in the endoplasmic reticulum was a prominent feature between the indicated groups (Fig. S2E, F). A heatmap of RNA-seq data revealed differential expression of ferroptosis, iron homeostasis, endoplasmic reticulum stress, and serine metabolism-related genes occur in AD tissue and RSL3-induced HASMCs. (Fig. 4C). To validate these findings, the mRNA and protein levels were assessed and we observed a consistent increase in the expression of NFE2L2 and HMOX1 during RSL3-induced ferroptosis, which was attenuated by SIL (Fig. 4D, E). The immunofluorescence image revealed that the nuclear translocation of NRF2 was increased in the RSL3 group, suggesting that NRF2 was activated. In contrast, in the SIL treatment (RSL3 + SIL) group, the translocation was decreased (Fig. 4F). Therefore, we deduced the NRF2/HMOX1 signal pathway regulated the process of the ferroptosis in HASMCs. The Lentivirus vector-mediated NFE2L2 overexpression of HASMCs was constructed. Then the mRNA of NRF2 was detected, and we found that *NFE2L2* significantly upregulated in the OE-NRF2 group (Fig. S3A). Western blot also showed that the NRF2 and HMOX1 were significantly increased in the OE-NRF2 group (Fig. 4G, H). Furthermore, the immunoblots analysis demonstrated that overexpression of NRF2 can inhibit the ferroptosis to some extent, while treatment with SIL significantly rescued the ferroptosis **(**Fig. 4I**)**. NRF2 is a nuclear transcription factor that plays a pivotal role in regulating cellular resistance to oxidative stress responses [29]. Our results showed that OE-NRF2 significantly inhibited the accumulation of lipid peroxidation and ROS in HASMC (Fig. 4J, K). OE-NRF2 also rescued the decrease of mitochondrial membrane potential which was induced by the RSL3 in the HASMCs (Fig. 4L). This is related to the fact that NRF2 is a major antioxidant transcription factor. The findings indicated that oxidative stress, particularly the activation of endoplasmic reticulum stress, plays a pivotal role in the induction of ferroptosis. Furthermore, the administration of SIL was found to significantly reverse ferroptosis and its associated oxidative stress in HASMCs.

### Silibinin is directly bound to HMOX1 to inhibit ferroptosis in HASMCs

**Fig. 5:**
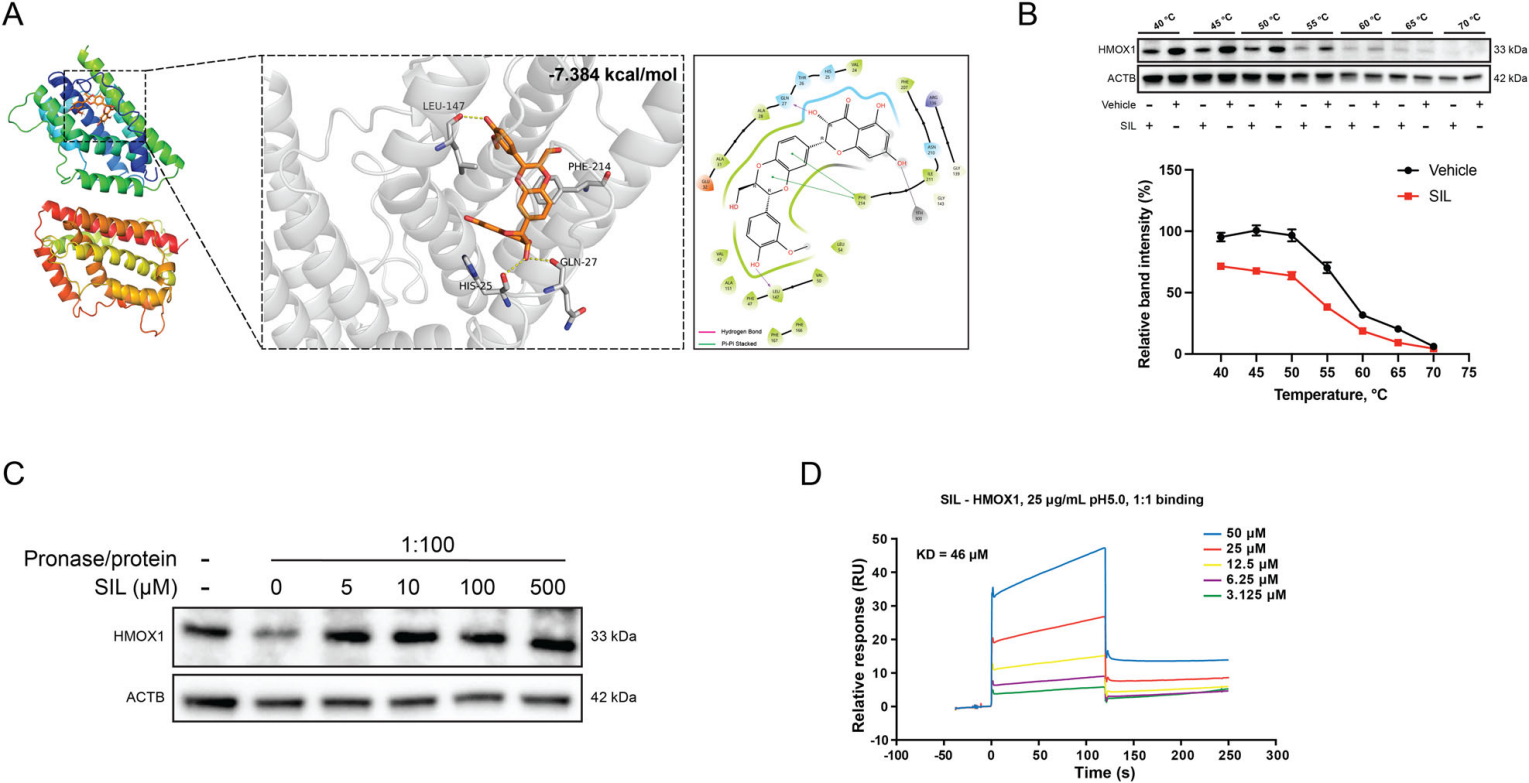
SIL is directly bound to HMOX1 to inhibit ferroptosis in HASMCs. **A** Chemical structure and in silico docking of SIL into the active pocket of human HMOX1 protein. **B** CETSA of HMOX1 in the absence and presence of SIL at different temperatures, the results were evaluated by western blots. **C** The direct interactions of SIL with HMOX1 were detected by using a DARTS assay. A ratio of Pronase E to the cell lysates is 1:100. The protein levels of HMOX1 were determined by western blot. **D** The affinity between HMOX1 protein and SIL (3.125–50 μM) was 46 μM (Ka = 7.66e + 02 1/Ms, Kd= 3.52e − 02 1/s).

To further clarify the specific mechanism of SIL regulation of HMOX1, molecular docking predictions indicated that SIL exhibits prominent binding sites with HMOX1. The estimated free energy of SIL binding to HMOX1 was -7.384 kcal/mol, suggesting a high level of binding activity. For the interaction, hydrogen bonds form at residues GLN 27 and LEU 147, and Pi-Pi Stacking occurs at residues PHE 214 (Fig. 5A). The direct interactions of SIL with HMOX1 were identified by the cellular thermal shift assays (CETSA), drug affinity responsive target stability (DARTS), and surface plasmon resonance (SPR). The CETSA results demonstrated that the melting curves of HMOX1 exhibited a significant shift at different temperatures (40, 45, 50, 55, 60, 65, and 70 °C) in the presence or absence of SIL treatment (Fig. 5B). The DARTS assay showed that treated with SIL can protect HMOX1 against the Pronase E (Fig. 5C). The SPR assay showed that the affinity between HMOX1 protein and SIL (3.125–50 μM) was 46 μM (Ka= 7.66e + 02 1/Ms, Kd= 3.52e-02 1/s) (Fig. 5D). Thus, SIL is a direct binding partner of HMOX1. Taken together, SIL is directly bound to HMOX1 to inhibit ferroptosis in HASMCs.

### Silibinin regulated dysfunction of iron homeostasis via inhibition of HMOX1

**Fig. 6:**
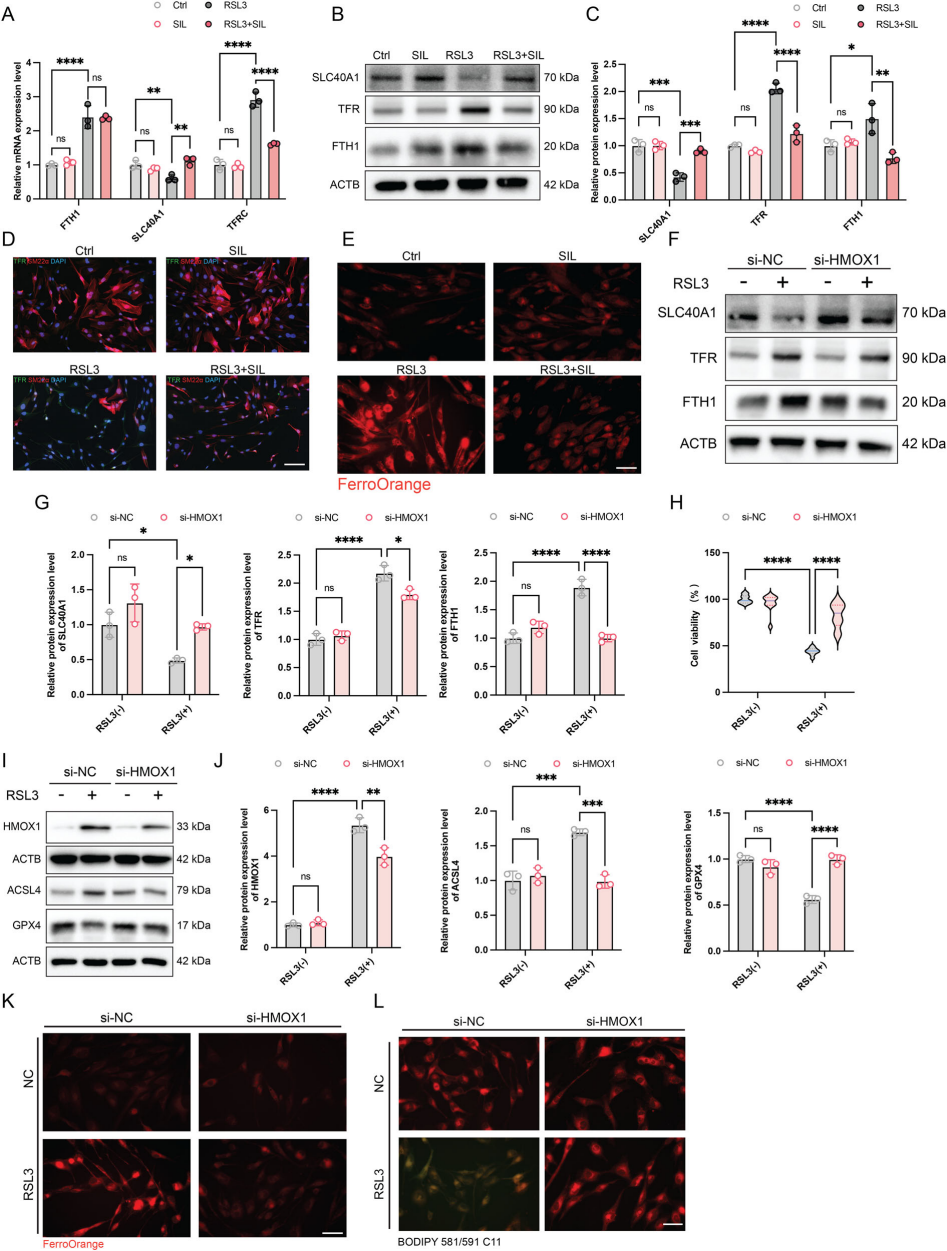
SIL alleviated dysfunction of iron homeostasis via inhibition of HMOX1. **A** Quantification of the mRNA levels of *FTH1, SLC40A1*, and *TFRC*. *n* = 3. **B**, **C** Immunoblots analysis of the protein expression of FTH1, SLC40A1, and TFR, *n* = 3. **D** Immunofluorescence staining for TFR. Scale bar, 50 μm. **E** Fluorescence staining images of Fe^2+^ in RSL3-induced HASMCs via FerroOrange probe. Scale bar, 50 μm. **F**–**L** The HASMCs were transfected with a siNC, and siHMOX1 for 48 h, then treated with RSL3 for 12 h. **F**, **G** The protein expression levels of FTH1, SLC40A1, and TFRC, *n* = 3. **H** Cell viability assay by CCK8, *n* = 10. **I**, **J** The protein expression levels of GPX4, ACSL4, and HMOX1, *n* = 3. **K** Representative fluorescence staining images of Fe^2+^ via FerroOrange probe. Scale bar, 50 μm. **L** Representative lipid ROS staining images. Scale bar, 50 μm. Data are presented as mean ± SD. *p < 0.05, **p < 0.01, ***p < 0.001, ****p < 0.0001; ns no significant difference.

Heme oxygenase 1 (HMOX1) is a heme-degrading enzyme that catalyzes the conversion of heme into biliverdin, a process that releases ferrous ions and carbon monoxide [30]. RNA-seq results showed that intracellular iron homeostasis marker (*SLC40A1, TFR*, and *FTH1*) was aberrantly expressed in the RSL3-induced HASMCs (Fig. 4C). To validate these findings, mRNA and protein levels were quantified. We found that TFR and FTH1 expression was elevated, while SLC40A1 expression was reduced in RSL3-induced ferroptosis. However, treatment with SIL abrogated the dysfunction of iron homeostasis (Fig. 6A–C). The immunofluorescence staining indicated that the expression level of TFR was increased in the RSL3 group, by contrast, in the SIL treatment (RSL3 + SIL) group, the expression level was decreased. Meanwhile, the expression of sm22α (the SMC marker) was decreased in the RSL3 treatment, while SIL treatment rescued this phenotype (Fig. 6D). In addition, our findings indicated that Fe^2+^, which is an inducer of ferroptosis, was significantly increased in RSL3-induced HASMCs. Nevertheless, the administration of the SIL resulted in a significant inhibition of Fe^2+^ accumulation in vitro (Fig. 6E). To further elucidate the potential mechanism between HMOX1 and iron homeostasis, we treated the HASMCs with siRNA (siNC and siHMOX1) and analyzed the expression of HMOX1, SLC40A1, TFR, and FTH1, cell viability, and the ferrous ion level. The Immunoblots analysis showed that siHMOX1 significantly inhibits the expression of HMOX1 (Fig. S3B). Notably, siHMOX1 rescued the aberrant expression of the iron homeostasis-related genes (SLC40 A1, TFR, and FTH1) (Fig. 6F, G). In addition, siHMOX1 improved the cell viability of the HASMCs induced by the RSL3 (Fig. 6H). Moreover, siHMOX1 was found to restore the protein levels of RSL3-induced ferroptosis-related genes (Fig. 6I, J), while simultaneously inhibiting the intracellular accumulation of Fe^2+^ (Fig. 6K) and lipid peroxidation levels (Fig. 6L). Taken together, these findings indicate that SIL alleviated dysfunction of iron homeostasis via inhibition of HMOX1, which is against ferroptosis.

### ERS plays a vital role in regulating serine metabolism in HASMCs ferroptosis

**Fig. 7:**
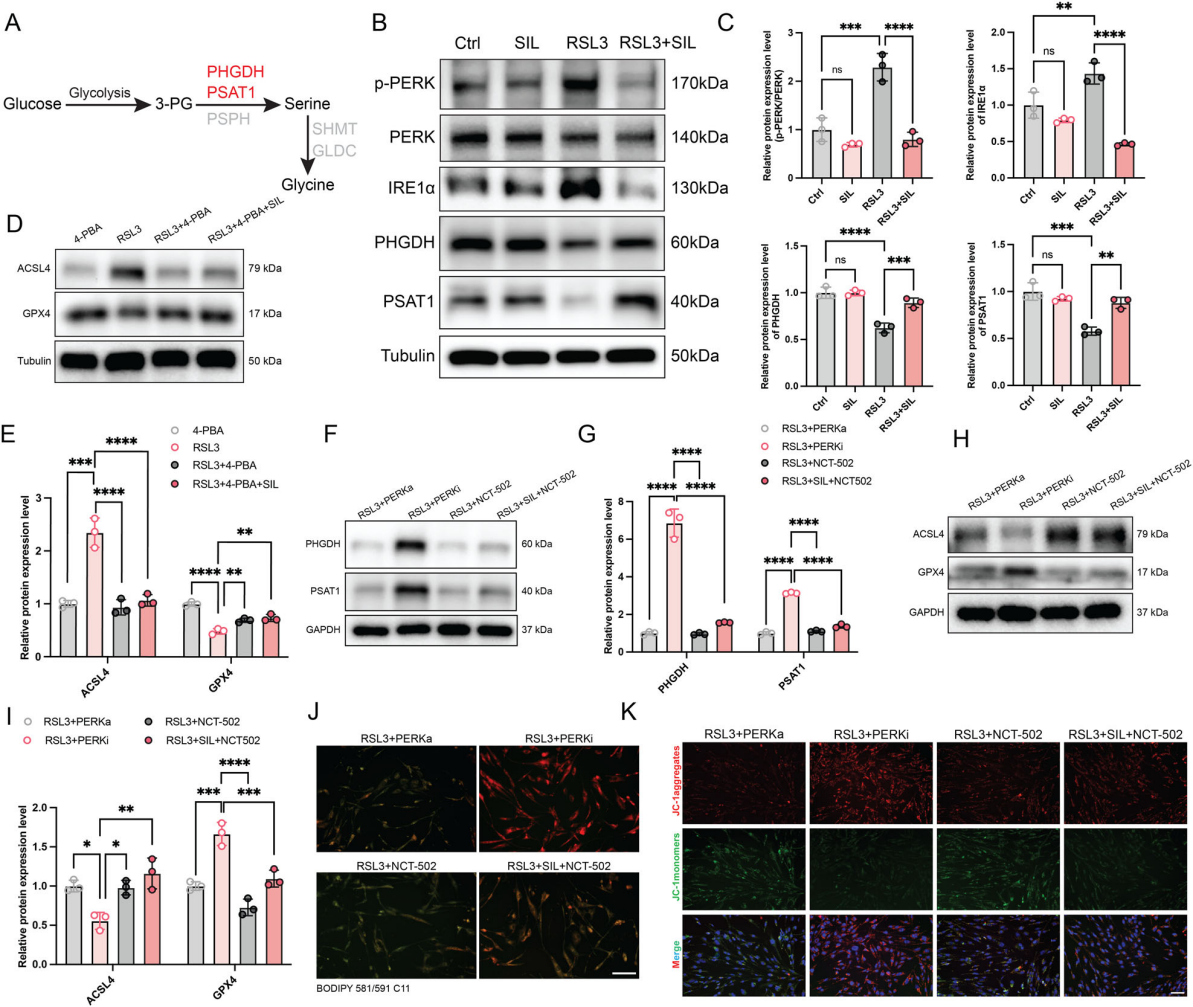
SIL rescued ferroptosis-induced dysfunction of ERS. **A** Pattern diagram of serine metabolism. **B**, **C** Western blot and relative gray value quantitative analysis of p-PERK, PERK, IRE1α, PHGDH, and PSAT1, *n* = 3. **D**, **E** The HASMCs were pretreated with 4-PBA (5 mM) and SIL (10 μM) in the indicated group for 24 h, followed by incubation with RSL3 for 12 h. The protein expression levels of GPX4 and ACSL4 were assayed by western blot in the indicated groups, *n* = 3. **F**–**K** HASMCs were pretreated with NCT-502 (PHGDH inhibitor, 5 μM), GSK2606414(PERK inhibitor, 1 μM), CCT020312(PERK activator,10 μM), and SIL (10 μM) in the indicated group for 24 h, followed by incubation with RSL3 for 12 h. **F**, **G** Western blot analysis of PHGDH and PSAT1 protein expression levels, *n* = 3. **H**, **I** The protein expression levels of GPX4 and ACSL4 in the indicated groups, *n* = 3. **J** The representative lipid ROS staining images. Scale bar, 50 μm. **K** Representative JC-1 staining images. Scale bar, 50 μm. Data are presented as mean ± SD. **p < 0.01, ***p < 0.001, ****p < 0.0001; ns: no significant difference.

To further illustrate the mechanism of ERS in regulating serine metabolism in the HASMCs ferroptosis, the pathway of serine metabolism is shown in Fig. 7A. According to RNA-seq results, we found that the key enzyme (PHGDH and PSAT1) that catalyzes the synthesis of serine was aberrantly expressed in the RSL3-induced HASMCs and AD aorta tissue. Our results demonstrated that the protein expression level of p-PERK, PERK, and IRE1α were significantly upregulated and PHGDH and PSAT1 were significantly downregulated in the RSL3 groups, and SIL administration significantly improved the expression of PHGDH and PSAT1 and inhibited the expression of p-PERK and IRE1α (Fig. 7B, C). To further investigate the relative signal mechanisms of ERS in RSL3-induced HASMCs ferroptosis, the ERS inhibitor 4-PBA, PERK inhibitor GSK2606414, PERK activator CCT020312, and PHGDH inhibitor NCT-502 were added. Western blot results showed that abnormal ACSL4 and GPX4 expression were remarkably rescued by the 4-PBA treatment (4-PBA + RSL3) group (Fig. 7D, E). The serine metabolism signaling was significantly inhibited in RSL3-induced HASMCs when ERS was activated using the CCT020312. In contrast, the GSK2606414 inhibited ERS and reversed the adverse effects (Fig. 7F, G). Western blot analysis demonstrated that ERS activator exacerbated RSL3-induced ferroptosis, whereas inhibition of ERS had the opposite effect (Fig. 7H, I). Notably, inhibition of serine metabolism using NCT-502 impeded the protective effect of SIL against RSL3-induced ferroptosis (Fig. 7H–K). These findings suggest that SIL inhibited ferroptosis by regulating serine metabolism in RSL3-induced HASMCs.

### Regulation of iron homeostasis and ERS attenuated the ferroptosis in the BAPN-induced AD mice

**Fig. 8:**
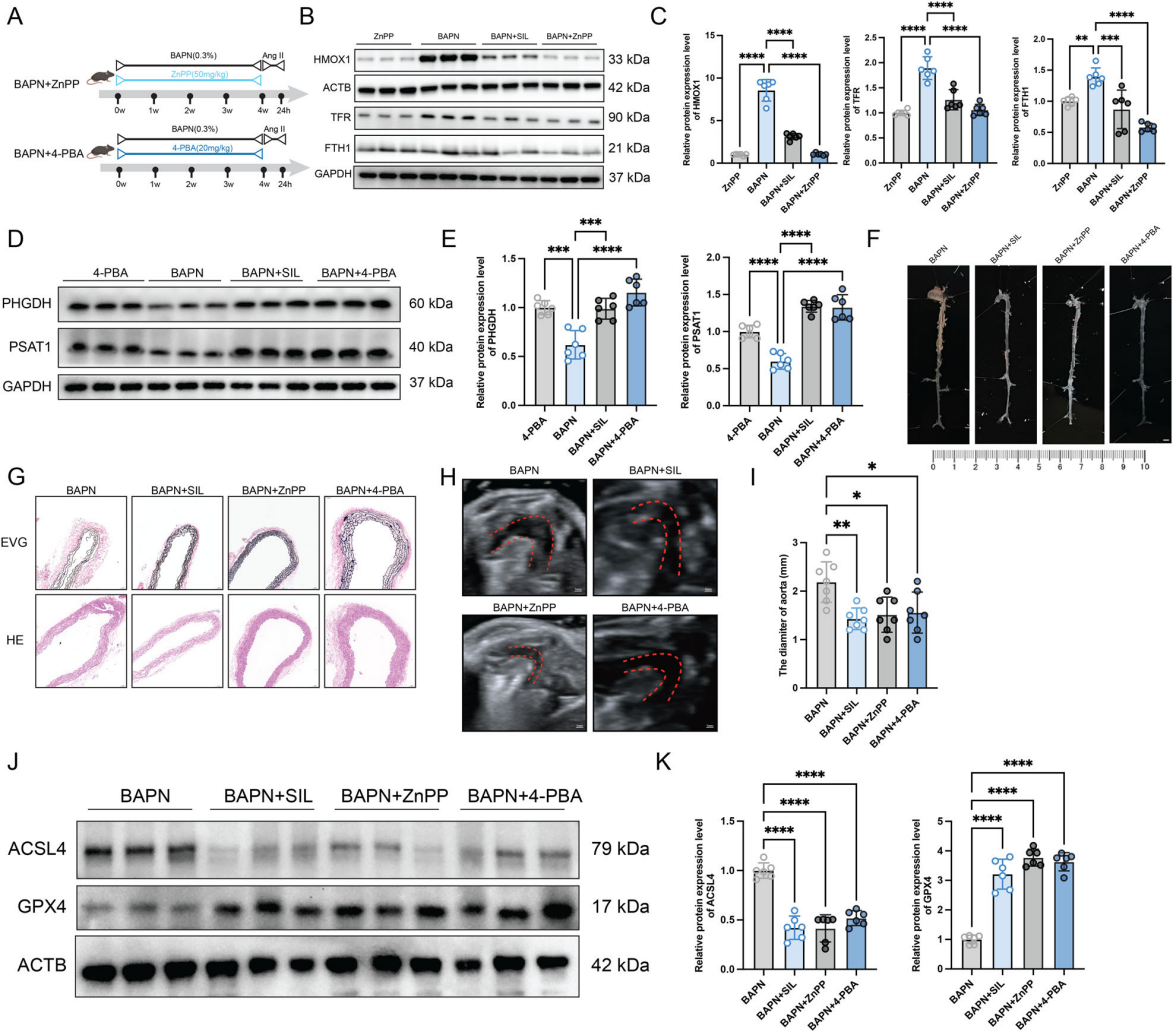
Regulation of iron homeostasis and ERS attenuated the ferroptosis in the BAPN-induced AD mice. **A** Schematic diagram depicting the animal treatment procedure. **B** Western blot analysis of HMOX1, FTH1, and TFR. **C** Relative quantitative analysis of the gray value, *n* = 6. **D** Western blot analysis of PHGDH and PSAT1. **E** Relative quantitative analysis of the gray value, *n* = 6. **F** Representative morphology of the mice aorta. Scale bar, 2 mm. **G** H&E staining and EVG staining images of mice aorta tissue, *n* = 6. **H** Representative ultrasound images of the mice aorta. **I** The diameter of the aorta, *n* = 6. **J**, **K** Immunoblot analysis of GPX4 and ACSL4 protein expression levels in the indicated groups, *n* = 6. Data are presented as mean ± SD. **p < 0.01, ***p < 0.001, ****p < 0.0001.

To further clarify the mechanisms by which SIL regulates iron homeostasis and ERS in vivo, ZnPP (an HO-1 inhibitor) and 4-PBA (an ERS inhibitor) were introduced (Fig. 8A). The expression levels of HMOX1, TFR, and FTH1 were significantly inhibited by ZnPP administrated in BAPN-induced AD mice (Fig. 8B, C). Meanwhile, 4-PBA treatment significantly reduced key protein expression levels of serine metabolic signaling (PHGDH and PSAT1) in BAPN-induced AD mice (Fig. 8D, E). BAPN-induced aortic dilation and dissection in mice, whereas treatment with ZnPP or 4-PBA attenuated this aortic dilation (Fig. 8F). Pathological staining demonstrated that the disorder and disruption of the elastin and dissection of the aorta, which were induced by BAPN, were significantly reversed by ZnPP or 4-PBA treatment (Fig. 8G). Ultrasound showed that there was significant dilatation of the aorta in the BAPN group, while treatment with ZnPP or 4-PBA reversed the adverse effect (Fig. 8H, I). Notably, immunoblots showed that both ZnPP and 4-PBA inhibited BAPN-induced ferroptosis in vivo (Fig. 8J, K).

## Discussion

In the current study, we observed dysregulated expression of ferroptosis-related molecules, including ACSL4, GPX4, and HMOX1, alongside elevated lipid peroxidation levels in the aorta of AD patients, highlighting the significance of ferroptosis in AD pathology. Additionally, we demonstrated SIL’s ability to attenuate ferroptosis in IKE or RSL3-induced HASMCs and BAPN-induced AD mice. RNA-sequencing analysis revealed RSL3-induced ferroptosis triggered dysfunction in iron homeostasis and endoplasmic reticulum stress in HASMCs. Mechanistically, SIL directly targeted HMOX1 to regulate iron homeostasis and inhibit ferroptosis in HASMCs. Interestingly, SIL also mitigated endoplasmic reticulum stress and restored serine metabolism in AD. Finally, SIL significantly inhibited aortic rupture and dissection formation, reducing ferroptosis in HASMCs in the BAPN-induced mice. Overall, our findings indicate that SIL protects against HASMCs ferroptosis and holds promise as a potential intervention for AD, as illustrated in the Graphical abstract.

Aortic dissection (AD) presents a significant cardiovascular threat with high mortality rates. Currently, percutaneous or open surgical interventions stand as the most effective means to mitigate the risk of aortic rupture [31]. Nevertheless, there is currently no evidence-based pharmacological intervention that has been proven to be effective. SMCs loss and defects are the major determinants of aortic diseases and AD. Ferroptosis is an iron-dependent, lipid peroxidation-driven cell death cascade that is associated with the pathology of AD [16, 32, 33]. The process of ferroptosis is initiated by the accumulation of reactive oxygen species (ROS), which in turn leads to severe lipid peroxidation. This is accompanied by iron overload and a reduction in the expression of antioxidant systems, including glutathione (GSH) and glutathione peroxidase 4 (GPX4) [34]. Recent studies have demonstrated that the inhibition of lipid peroxidation and ferroptosis has the potential to reduce morbidity and mortality and attenuate aortic dilation in BAPN-induced mice [16]. SP2509, a novel ferroptosis inhibitor, could alleviate intracellular Fe^2+^ accumulation, thus preventing oxidative stress and ferroptosis in the SMCs [32]. The understanding of ferroptosis regulatory mechanisms in AD remains limited in the literature, and its therapeutic potential has yet to be fully explored. This presents a promising avenue for further investigation into the role of ferroptosis in AD.

SIL is the naturally occurring flavonoid extracted from the seeds of *S. marianum*, and its cardioprotective effect has been recently recognized [25–27]. It has been demonstrated that SIL can directly target ACSL4, thereby inhibiting lipid peroxidation and preventing ferroptosis in HepG2 cells [28]. In this study, we also found that the ferroptosis marker (ACSL4, GPX4, HMOX1, and 4-HNE) had aberrant expression in the human AD aorta tissue and BAPN-induced mice aorta. These findings align with previous research indicating the involvement of ferroptosis in AD [16]. To determine whether SIL can exert a protective effect against ferroptosis in AD, the mice were administered with SIL intraperitoneally. Our data demonstrated that SIL reduced the incident of rupture and dissection in BAPN-induced mice by alleviating aortic structure damage, mitochondrial ultrastructural damage, and lipid peroxidation, as well as inhibiting ferroptosis in vivo.

Although the human aorta contains a multitude of cell types, vascular smooth muscle cells (SMCs) of disparate developmental origins represent the predominant cell type that forms the vascular wall. Therefore, we used the RSL3 or IKE-induced HASMCs to build a classical ferroptosis cell model. In our present study, we observed that RSL3 or IKE-induced ferroptosis in HASMCs in vitro, while SIL exhibited protective effects by enhancing cell viability, reducing lipid peroxidation, restoring mitochondrial function, and suppressing ferroptosis. However, the precise mechanism underlying SIL’s impact on the occurrence and progression of AD remains incompletely understood. To elucidate the potential molecular mechanisms, we conducted RNA sequencing and identified that SIL significantly reversed the abnormal gene expression induced by RSL3. A total of 57 DEGs regulated by SIL were subjected to Hub gene enrichment analysis, which identified HMOX1 as the core gene. Additionally, Gene Ontology (GO) and Gene Set Enrichment Analysis (GSEA) enrichment analysis revealed that these DEGs are closely associated with oxidative stress and serine metabolic processes. Consequently, it is postulated that SIL may exert a protective effect by regulating HMOX1 expression, oxidative stress, and serine metabolism processes.

The nuclear factor erythroid 2-related factor 2 (NRF2) serves as a crucial regulator of the cellular antioxidant response, governing the expression of genes involved in counteracting oxidative and electrophilic stresses. Evidence suggests that the NRF2 signaling pathway, which regulates lipid peroxidation and ferroptosis, is involved in cardiovascular diseases [29, 35–37]. Therefore, we speculated that SIL exerts its effects by regulating the NRF2/HMOX1 signaling pathway. Intriguingly, we observed that OE-NRF2 partially reversed RSL3-induced ferroptosis in HASMCs, whereas SIL treatment significantly reversed ferroptosis. These findings suggest that the protective effect of NRF2 may be related to its modulation of oxidative stress signaling. Interestingly, the molecular docking analysis revealed that SIL exhibited a high degree of affinity for the binding sites of HMOX1. The interaction between SIL and HMOX1 is mediated by the formation of hydrogen bonds at residues GLN 27 and LEU 147, and by Pi-Pi Stacking at residues PHE 214. We also identified the direct interactions between SIL and HMOX1 in vitro using CETSA, DARTS, and SPR assays. These findings align with previous observations that identified HMOX1 as a potential target of SIL in living cells through the Living Cell-Target Responsive Accessibility Profiling (LC-TRAP) approach [28]. Heme oxygenase 1 serves as an indicator of oxidative stress and is capable of oxidizing heme to free ferrous iron, biliverdin, and CO [38]. There is still controversy about the role of HMOX1 in regulating ferroptosis [11, 39, 40]. Multiple lines of research demonstrate that the activation of HMOX1 protects cells from ferroptosis by reducing oxidative stress [41–43]. However, in line with our current findings, a recent study demonstrated that induction of HMOX1 is linked to the severity of disease in human abdominal aortic aneurysms [44]. It also has proven that inhibition of HMOX1 alleviates BAPN-induced TAA in mice by restoring the extracellular matrix (ECM) [45]. One potential explanation for this phenomenon is that the degree of HMOX1 activation determines whether it exerts a stimulatory or inhibitory effect on ferroptosis. The maintenance of intracellular iron homeostasis relies on the coordinated actions of three key proteins: SLC40A1, which exports iron; TFR, which imports iron; and FTH1, which stores iron [39, 46]. To elucidate the protective mechanism of inhibiting HMOX1, the altered expression levels of HMOX1, SLC40A1, FTH1, and TFR in RSL3-induced HASMCs were subjected to more rigorous investigation. We found that siHMOX1 can restore intracellular iron homeostasis and then alleviate the ferroptosis of HASMCs. These findings align with earlier observations that ZnPP (an HMOX1 inhibitor) blocks retinal pigment epithelium ferroptosis by inhibiting HMOX1 expression and accumulation of free ferrous iron [46].

The results of RNA sequencing analysis indicated that endoplasmic reticulum stress signaling plays another pivotal role in the ferroptosis of HASMCs regulated by SIL. Therefore, the expression of ERS-related proteins (p-PERK, PERK, and IRE1α) was determined. We found that SIL significantly inhibited the expression levels of ERS proteins. Despite a recent study showed that PERK activation mediated the elevation of phosphoserine aminotransferase 1 (PSAT1) and serine biosynthesis via the downstream transcription factor ATF-4 in macrophages [47]. However, our data demonstrated that SIL significantly increased the expression levels of PHGDH and PAST1 in RSL3-induced HASMCs. To further clarify the relationship between SIL regulation of ERS and serine metabolism, the 4-PBA (ERS inhibitor), GSK2606414(PERK inhibitor), CCT020312(PERK activator), and NCT-502(PHGDH inhibitor) were employed. We found that the use of 4-PBA and GSK2606414 significantly inhibited RSL3-induced ferroptosis, while treatment with NCT-502 reversed the protective effect of SIL against ferroptosis in vitro. The increased biosynthesis of serine leads to improved mitochondrial function and increased glycine production, thereby contributing to cellular metabolism and maintaining the balance of oxygen-free radical metabolism in the body [47]. This is consistent with our findings that SIL modulates ER stress, which in turn affects serine metabolism, against ferroptosis in RSL3-induced HASMCs.

Finally, to clarify the protective effects of inhibition of ERS as well as HMOX1 in vivo, the BAPN-induced mice were treated with 4-PBA and ZnPP. Our findings indicated that both significantly inhibited aortic dilatation and dissection, and attenuated ferroptosis in vivo. These findings align with prior reports that ZnPP alleviates BAPN-induced TAA in mice by regulating macrophage phenotype and suppressing the expression of matrix metalloproteinases (MMPs) [45].

Despite the aforementioned findings, our research still has some limitations, including 1) The aorta harbors a variety of cell types, and in the future, we will further define the regulatory role of SIL on other cell types, such as fibroblasts and macrophages. 2) Although Animal models have provided key insights into the pathophysiological mechanism of AD. The disparity observed across species underscores the necessity for a more predictive disease model based on human cells to better understand the pathogenesis of AD. Bioengineered blood vessels established based on AD patient-derived hiPSC will provide a good research platform to further clarify the role of SIL in protecting against AD [48]. 3) There are still some gene expression differences in RNA-sequencing between Human tissue and RSL3-induced HASMCs. This is constrained by the analysis of end-stage human AD aortic tissue, which undergoes compensatory molecular and cellular changes during the progression of aneurysm and dissection.

## Supplementary information

Supplementary Material

Western Blot raw data

## Data Availability

All data generated during this study are included in this published article and its Supplementary Information files.

## References

[1] Erbel R, Aboyans V, Boileau C, Bossone E, Bartolomeo RD, Eggebrecht H, et al. ESC Guidelines on the diagnosis and treatment of aortic diseases: Document covering acute and chronic aortic diseases of the thoracic and abdominal aorta of the adult. The Task Force for the Diagnosis and Treatment of Aortic Diseases of the European Society of Cardiology (ESC). Eur Heart J. 2014;35:2873–926. 25173340 10.1093/eurheartj/ehu281

[2] Olsson C, Thelin S, Ståhle E, Ekbom A, Granath F. Thoracic aortic aneurysm and dissection: increasing prevalence and improved outcomes reported in a nationwide population-based study of more than 14,000 cases from 1987 to 2002. Circulation. 2006;114:2611–8. 17145990 10.1161/CIRCULATIONAHA.106.630400

[3] Isselbacher EM, Preventza O, Hamilton Black J 3rd, Augoustides JG, Beck AW, Bolen MA, et al. ACC/AHA guideline for the diagnosis and management of aortic disease: a report of the American Heart Association/American College of Cardiology Joint Committee on Clinical Practice Guidelines. Circulation. 2022;146:e334–e482. 36322642 10.1161/CIR.0000000000001106 PMC9876736

[4] Lindsay J Jr, Hurst JW. Clinical features and prognosis in dissecting aneurysm of the aorta. A re-appraisal. Circulation. 1967;35:880–8. 6021777 10.1161/01.cir.35.5.880

[5] Olsson C, Ahlsson A, Fuglsang S, Geirsson A, Gunn J, Hansson EC, et al. Medium-term survival after surgery for acute Type A aortic dissection is improving. Eur J Cardiothorac Surg. 2017;52:852–7. 28957999 10.1093/ejcts/ezx302

[6] Chen Y, He Y, Wei X, Jiang DS. Targeting regulated cell death in aortic aneurysm and dissection therapy. Pharm Res. 2022;176:106048. 10.1016/j.phrs.2021.106048 34968685

[7] Li N, Yi X, He Y, Huo B, Chen Y, Zhang Z, et al. Targeting ferroptosis as a novel approach to alleviate aortic dissection. Int J Biol Sci. 2022;18:4118–34. 35844806 10.7150/ijbs.72528 PMC9274489

[8] Luo W, Wang Y, Zhang L, Ren P, Zhang C, Li Y, et al. Critical role of cytosolic DNA and its sensing adaptor STING in aortic degeneration, dissection, and rupture. Circulation. 2020;141:42–66. 31887080 10.1161/CIRCULATIONAHA.119.041460 PMC6939474

[9] Zhang W, Wang M, Gao K, Zhong X, Xie Y, Dai L, et al. Pharmacologic IRE1α kinase inhibition alleviates aortic dissection by decreasing vascular smooth muscle cells apoptosis. Int J Biol Sci. 2022;18:1053–64. 35173538 10.7150/ijbs.63593 PMC8771832

[10] Xia L, Sun C, Zhu H, Zhai M, Zhang L, Jiang L, et al. Melatonin protects against thoracic aortic aneurysm and dissection through SIRT1-dependent regulation of oxidative stress and vascular smooth muscle cell loss. J Pineal Res. 2020;69:e12661. 32329099 10.1111/jpi.12661

[11] Fang X, Wang H, Han D, Xie E, Yang X, Wei J, et al. Ferroptosis as a target for protection against cardiomyopathy. Proc Natl Acad Sci USA. 2019;116:2672–80. 30692261 10.1073/pnas.1821022116 PMC6377499

[12] Qi Z, Liu R, Ju H, Huang M, Li Z, Li W, et al. microRNA-130b-3p attenuates septic cardiomyopathy by regulating the AMPK/mTOR signaling pathways and directly targeting ACSL4 against ferroptosis. Int J Biol Sci. 2023;19:4223–41. 37705752 10.7150/ijbs.82287 PMC10496507

[13] Park TJ, Park JH, Lee GS, Lee JY, Shin JH, Kim MW, et al. Quantitative proteomic analyses reveal that GPX4 downregulation during myocardial infarction contributes to ferroptosis in cardiomyocytes. Cell Death Dis. 2019;10:835. 31685805 10.1038/s41419-019-2061-8 PMC6828761

[14] Mancardi D, Mezzanotte M, Arrigo E, Barinotti A, Roetto A. Iron overload, oxidative stress, and ferroptosis in the failing heart and liver. Antioxidants (Basel). 2021;10:1864. 34942967 10.3390/antiox10121864 PMC8698778

[15] Liu B, Zhao C, Li H, Chen X, Ding Y, Xu S. Puerarin protects against heart failure induced by pressure overload through mitigation of ferroptosis. Biochem Biophys Res Commun. 2018;497:233–40. 29427658 10.1016/j.bbrc.2018.02.061

[16] Chen Y, Yi X, Huo B, He Y, Guo X, Zhang Z, et al. BRD4770 functions as a novel ferroptosis inhibitor to protect against aortic dissection. Pharm Res. 2022;177:106122. 10.1016/j.phrs.2022.106122 35149187

[17] Xie Y, Hou W, Song X, Yu Y, Huang J, Sun X, et al. Ferroptosis: process and function. Cell Death Differ. 2016;23:369–79. 26794443 10.1038/cdd.2015.158 PMC5072448

[18] Liao M, Liu Z, Bao J, Zhao Z, Hu J, Feng X, et al. A proteomic study of the aortic media in human thoracic aortic dissection: implication for oxidative stress. J Thorac Cardiovasc Surg. 2008;136:65–72. 72.e61-63. 18603055 10.1016/j.jtcvs.2007.11.017

[19] Wellington K, Jarvis B. Silymarin: a review of its clinical properties in the management of hepatic disorders. BioDrugs. 2001;15:465–89. 11520257 10.2165/00063030-200115070-00005

[20] Tuli HS, Mittal S, Aggarwal D, Parashar G, Parashar NC, Upadhyay SK, et al. Path of Silibinin from diet to medicine: A dietary polyphenolic flavonoid having potential anti-cancer therapeutic significance. Semin Cancer Biol. 2021;73:196–218. 33130037 10.1016/j.semcancer.2020.09.014

[21] Pradhan SC, Girish C. Hepatoprotective herbal drug, silymarin from experimental pharmacology to clinical medicine. Indian J Med Res. 2006;124:491–504. 17213517

[22] Liu Y, Xu W, Zhai T, You J, Chen Y. Silibinin ameliorates hepatic lipid accumulation and oxidative stress in mice with non-alcoholic steatohepatitis by regulating CFLAR-JNK pathway. Acta Pharm Sin B. 2019;9:745–57. 31384535 10.1016/j.apsb.2019.02.006 PMC6664044

[23] Rašković A, Stilinović N, Kolarović J, Vasović V, Vukmirović S, Mikov M. The protective effects of silymarin against doxorubicin-induced cardiotoxicity and hepatotoxicity in rats. Molecules. 2011;16:8601–13. 21993249 10.3390/molecules16108601 PMC6264541

[24] Li Volti G, Salomone S, Sorrenti V, Mangiameli A, Urso V, Siarkos I, et al. Effect of silibinin on endothelial dysfunction and ADMA levels in obese diabetic mice. Cardiovasc Diabetol. 2011;10:62. 21756303 10.1186/1475-2840-10-62 PMC3152512

[25] Anestopoulos I, Kavo A, Tentes I, Kortsaris A, Panayiotidis M, Lazou A, et al. Silibinin protects H9c2 cardiac cells from oxidative stress and inhibits phenylephrine-induced hypertrophy: potential mechanisms. J Nutr Biochem. 2013;24:586–94. 22818713 10.1016/j.jnutbio.2012.02.009

[26] Wang X, Zhang Z, Wu SC. Health benefits of *Silybum marianum*: phytochemistry, pharmacology, and applications. J Agric Food Chem. 2020;68:11644–64. 33045827 10.1021/acs.jafc.0c04791

[27] Kadoglou NPE, Panayiotou C, Vardas M, Balaskas N, Kostomitsopoulos NG, Tsaroucha AK, Valsami G. A comprehensive review of the cardiovascular protective properties of silibinin/silymarin: a new kid on the block. Pharmaceuticals (Basel). 2022;15:538. 35631363 10.3390/ph15050538 PMC9145573

[28] Yan W, Wang D, Wan N, Wang S, Shao C, Zhang H, et al. Living cell-target responsive accessibility profiling reveals silibinin targeting ACSL4 for combating ferroptosis. Anal Chem. 2022;94:14820–6. 36260072 10.1021/acs.analchem.2c03515

[29] Dodson M, Castro-Portuguez R, Zhang DD. NRF2 plays a critical role in mitigating lipid peroxidation and ferroptosis. Redox Biol. 2019;23:101107. 30692038 10.1016/j.redox.2019.101107 PMC6859567

[30] Schipper HM, Song W, Zukor H, Hascalovici JR, Zeligman D. Heme oxygenase-1 and neurodegeneration: expanding frontiers of engagement. J Neurochem. 2009;110:469–85. 19457088 10.1111/j.1471-4159.2009.06160.x

[31] Nienaber CA, Clough RE. Management of acute aortic dissection. Lancet. 2015;385:800–11. 25662791 10.1016/S0140-6736(14)61005-9

[32] He Y, Wang X, Chen S, Luo H, Huo B, Guo X, et al. SP2509 functions as a novel ferroptosis inhibitor by reducing intracellular iron level in vascular smooth muscle cells. Free Radic Biol Med. 2024;219:49–63. 38608823 10.1016/j.freeradbiomed.2024.04.220

[33] Song W, Chen Y, Qin L, Xu X, Sun Y, Zhong M, et al. Oxidative stress drives vascular smooth muscle cell damage in acute Stanford type A aortic dissection through HIF-1α/HO-1 mediated ferroptosis. Heliyon. 2023;9:e22857. 38125409 10.1016/j.heliyon.2023.e22857 PMC10730757

[34] Bersuker K, Hendricks JM, Li Z, Magtanong L, Ford B, Tang PH, et al. The CoQ oxidoreductase FSP1 acts parallel to GPX4 to inhibit ferroptosis. Nature. 2019;575:688–92. 31634900 10.1038/s41586-019-1705-2 PMC6883167

[35] Zhang Q, Liu J, Duan H, Li R, Peng W, Wu C. Activation of Nrf2/HO-1 signaling: an important molecular mechanism of herbal medicine in the treatment of atherosclerosis via the protection of vascular endothelial cells from oxidative stress. J Adv Res. 2021;34:43–63. 35024180 10.1016/j.jare.2021.06.023 PMC8655139

[36] Wang X, Chen X, Zhou W, Men H, Bao T, Sun Y, et al. Ferroptosis is essential for diabetic cardiomyopathy and is prevented by sulforaphane via AMPK/NRF2 pathways. Acta Pharm Sin B. 2022;12:708–22. 35256941 10.1016/j.apsb.2021.10.005 PMC8897044

[37] Wang D, Wu J, Le S, Wang H, Luo J, Li R, et al. Oltipraz, the activator of nuclear factor erythroid 2-related factor 2 (Nrf2), protects against the formation of BAPN-induced aneurysms and dissection of the thoracic aorta in mice by inhibiting activation of the ROS-mediated NLRP3 inflammasome. Eur J Pharmacol. 2022;936:175361. 36336010 10.1016/j.ejphar.2022.175361

[38] Hassannia B, Vandenabeele P, Vanden Berghe T. Targeting ferroptosis to iron out cancer. Cancer Cell. 2019;35:830–49. 31105042 10.1016/j.ccell.2019.04.002

[39] Chang LC, Chiang SK, Chen SE, Yu YL, Chou RH, Chang WC. Heme oxygenase-1 mediates BAY 11-7085 induced ferroptosis. Cancer Lett. 2018;416:124–37. 29274359 10.1016/j.canlet.2017.12.025

[40] Menon AV, Liu J, Tsai HP, Zeng L, Yang S, Asnani A, et al. Excess heme upregulates heme oxygenase 1 and promotes cardiac ferroptosis in mice with sickle cell disease. Blood. 2022;139:936–41. 34388243 10.1182/blood.2020008455 PMC8832481

[41] Adedoyin O, Boddu R, Traylor A, Lever JM, Bolisetty S, George JF, et al. Heme oxygenase-1 mitigates ferroptosis in renal proximal tubule cells. Am J Physiol Ren Physiol. 2018;314:F702–f714. 10.1152/ajprenal.00044.2017 PMC6031916 28515173

[42] Yao D, Bao L, Wang S, Tan M, Xu Y, Wu T, et al. Isoliquiritigenin alleviates myocardial ischemia-reperfusion injury by regulating the Nrf2/HO-1/SLC7a11/GPX4 axis in mice. Free Radic Biol Med. 2024;221:1–12. 38734270 10.1016/j.freeradbiomed.2024.05.012

[43] Zhong X, Wang K, Wang Y, Wang L, Wang S, Huang W, et al. Angiotension II directly bind P2X7 receptor to induce myocardial ferroptosis and remodeling by activating human antigen R. Redox Biol. 2024;72:103154. 38626575 10.1016/j.redox.2024.103154 PMC11035111

[44] Hofmann A, Müglich M, Wolk S, Khorzom Y, Sabarstinski P, Kopaliani I, et al. Induction of heme Oxygenase-1 is linked to the severity of disease in human abdominal aortic aneurysm. J Am Heart Assoc. 2021;10:e022747. 34622673 10.1161/JAHA.121.022747 PMC8751892

[45] Song W, Shen K, Fu G, Qin L, Bagaber G, Chen J, et al. Inhibition of heme oxygenase 1 alleviates thoracic aortic aneurysm via restoration of extracellular matrix. Biochem Biophys Res Commun. 2024;694:149405. 38147696 10.1016/j.bbrc.2023.149405

[46] Tang Z, Ju Y, Dai X, Ni N, Liu Y, Zhang D, et al. HO-1-mediated ferroptosis as a target for protection against retinal pigment epithelium degeneration. Redox Biol. 2021;43:101971. 33895485 10.1016/j.redox.2021.101971 PMC8099560

[47] Raines LN, Zhao H, Wang Y, Chen HY, Gallart-Ayala H, Hsueh PC, et al. PERK is a critical metabolic hub for immunosuppressive function in macrophages. Nat Immunol. 2022;23:431–45. 35228694 10.1038/s41590-022-01145-x PMC9112288

[48] Yang Y, Feng H, Tang Y, Wang Z, Qiu P, Huang X, et al. Bioengineered vascular grafts with a pathogenic TGFBR1variant model aneurysm formation in vivo and reveal underlying collagen defects. Sci Transl Med. 2024;16:eadg6298. 38718134 10.1126/scitranslmed.adg6298 PMC11193908

