# Supplementary material for: Dual functions of silibinin in attenuating aortic dissection via regulating iron homeostasis and endoplasmic reticulum stress against ferroptosis
Source: Cell Death Dis. 2024 Dec 18;15(12):900. doi: 10.1038/s41419-024-07309-x (PMC11655547; doi:10.1038/s41419-024-07309-x)
Supplement: Supplementary file 1 — Supplementary Material [file 41419_2024_7309_MOESM1_ESM.docx]

**Dual Functions of Silibinin in Attenuating Aortic Dissection via Regulating Iron Homeostasis and Endoplasmic Reticulum Stress against Ferroptosis**

**Zhen Qi ^a, 1^,** **Qiu-Guo Wang ^a, 1^, Meng-Xi Huang ^b^, Yi-Fan Zeng ^a^, Jing-Yu Li ^a^, Zhi-Cheng Duan ^a^, Ling Tan ^a, *^, Hao Tang ^a, *^**

^a^ *Department of Cardiovascular Surgery,* *the Second Xiangya Hospital, Central South University, Changsha, China*

^b^ *Jinshan Hospital, Fudan University, Shanghai, China*

* Corresponding author.

E-mail address: dr.tanling@csu.edu.cn (Ling Tan), dr.tanghao@csu.edu.cn (Hao Tang).

^1^ These authors contributed equally to this study.

The PDF file includes:

Supplementary Materials and Methods

Fig. S1 to S3

Tables S1 to S3

**Supplementary Materials and Methods**

**Reagents**

Silibinin (SIL, S0417, ≥98% purity) was obtained from Sigma (St. Louis, MO, United States). Imidazole ketone erastin (IKE, S8877, ≥99.51% purity) was purchased from Selleck (Selleck Chemicals, United States). Zinc Protoporphyrin-9 (ZnPP, sc-200329, >95% purity) was obtained from Santa Cruz (Santa Cruz, United States). RSL3 (HY-100218A, ≥99.90% purity), GSK2606414 (HY-18072, ≥99.38% purity), CCT020312 (HY-119240, ≥99.28% purity), NCT-502 (HY-117240, ≥99.18% purity), Ferrostatin-1 (Fer-1, ≥99.72% purity), and 4-Phenylbutyric acid (4-PBA, HY-A0281, ≥99.98% purity) were obtained from MCE company (Brea, CA, United States).

**H&E and EVG Staining**

Aortic tissues from both patients and mice were fixed in 4% paraformaldehyde at room temperature for 24 hours and subsequently embedded in paraffin. Sections of 5-μm thickness were prepared and stained with hematoxylin and eosin (H&E) according to the manufacturer's instructions to assess aortic morphology. The degree of elastic fiber rupture was evaluated using Elastica van Gieson (EVG) staining.

**Cell Counting Kit-8 (CCK 8) Assay**

The Cell Counting Kit-8 (CCK-8) Assay Kit (GlpBio, CA, United States) was utilized to assess cell viability. In brief, 1 × 10^4 HASMCs/well were seeded into 96-well plates and incubated overnight. Upon reaching 70% confluence, cells were subjected to various experimental treatments. Subsequently, 10 μL of CCK-8 solution was added to each well, followed by incubation at 37°C for 2 hours. Absorbance was then measured at 450 nm.

**Reactive oxygen species assays**

The levels of reactive oxygen species (ROS) in HASMCs were determined using the ROS Assay Kit (Beyotime, Shanghai, China). Briefly, HASMCs were seeded in 12-well plates and treated with a ferroptosis inducer for the specified duration. Subsequently, 10 μM DCFH-DA was added and the cells were incubated for 20 minutes at 37°C, protected from light. After two washes with DPBS, the image of HASMCs was captured by fluorescence microscopy.

**Measurement of the** **Mitochondrial Membrane Potential (ΔΨm)**

The JC-1 Mitochondrial Membrane Potential Assay Kit (Beyotime, Shanghai, China) was employed to assess the mitochondrial membrane potential (MMP) of HASMCs, following the instructions provided by the manufacturer. In brief, HASMCs were washed twice with cold DPBS and then incubated with JC-1 working solution at 37°C for 30 minutes, protected from light. Following two washes with JC-1 washing buffer and subsequent labeling of cell nuclei with Hoechst 33342, fluorescence microscopy was employed to observe the images.

**Western Blotting**

HASMCs and aortic tissue samples were homogenized using RIPA lysis buffer supplemented with protease and phosphatase inhibitors (Beyotime Biotechnology, Shanghai, China). The protein concentration was quantified using the BCA Protein Assay Kit (Thermo Fisher Scientific, United States). Equal amounts of protein extract were separated by SDS-PAGE and transferred to PVDF membranes (Millipore, United States). After blocking with protein-free rapid blocking buffer (PS108P, EpiZyme, China) at room temperature for 20 minutes, the membranes were incubated with primary antibodies overnight at 4°C. Subsequently, the membranes were probed with the corresponding HRP-conjugated secondary antibodies (Cell Signaling Technology) for 1 hour at room temperature. Immunoreactive bands were visualized using the Omni-ECL™ Enhanced Pico Light Chemiluminescence Kit (SQ101L, EpiZyme, China), and densitometric analysis was performed using Image J software (NIH, MD, United States). The following primary antibodies were used at the indicated dilutions: ACSL4 (1:10000, Abcam, ab155282), GPX4 (1:10000, Abcam, ab125066), p-NRF2 (1:10000, Abcam, ab76026), p-NRF2 (1:1000, ABclonal, AP1133), NRF2 (1:1000, ABclonal, A0674), HMOX1 (1:1000, Abcam, ab68477), FTH1 (1:800, ABclonal, A19544), SLC40A1 (1:500, Proteintech, 26601-1-AP), TFRC (1:1000, Proteintech, 10084-2-AP), p-PERK(1:800, ABclonal, AP1501), PERK (1:1000, Proteintech, 24390-1-AP), PHGDH(1:1000, Proteintech, 14719-1-AP), PSAT1(1:1000, Proteintech, 10501-1-AP), GAPDH (1:1000, Cell Signaling Technology, 5174S), β-Actin (1:1000, Cell Signaling Technology, 4970S), Tubulin (1:1000, Cell Signaling Technology, 2144S).

**Quantitative real-time PCR (RT-qPCR)**

Total RNA was extracted from aortic tissue or HASMCs using TRIzol™ Reagent (Invitrogen, United States) following the manufacturer's instructions. Subsequently, cDNA was synthesized in a 20 μL reaction volume using the HiScript III RT SuperMix for qPCR (Vazyme, Nanjing, China) according to the manufacturer's protocol. Quantitative real-time PCR was conducted using ChamQ SYBR GREEN Color qPCR Master Mix (Vazyme, Nanjing, China). GAPDH was utilized as the reference gene for mRNA analysis. The relative expression levels of mRNA were determined using the 2^-ΔΔCT^ method. The primers sequencing information used in this research is provided in **Supplementary Table S3**.

**Drug affinity responsive target stability (****DARTS) assay**

The DARTS assay was conducted following previously described methods ^[29]^. HASMCs were washed twice with PBS, followed by the addition of M-PER lysis buffer for lysis on ice for 15 minutes. The cell lysates were then collected by centrifugation at 15,000 × g for 20 minutes. The supernatants were collected and quantified using a BCA protein assay kit (Thermo Fisher Scientific). Subsequently, the lysates were mixed with 10× TNC buffer and incubated with or without SIL at the specified concentrations at room temperature for 1 hour. Following the SIL treatment, Pronase E was added at the specified protease/protein ratio and digested for 30 minutes at 37°C. Subsequently, 5× loading buffer was added, and the samples were heated to 100°C for 15 minutes. Quantitative analysis was performed using Western Bolt.

**Surface plasmon resonance (SPR) assay**

The affinity between the HMOX1 protein and the small-molecule compound SIL was determined using the Biacore^TM^ 8K instrument (Cytiva, United States). The ligand protein was diluted to a concentration of 2 μg/mL using an acetic acid solution with pH values of 4.5, 5.0, and 5.5. A pH scouting test was conducted to determine the appropriate pH buffer for immobilizing the ligand protein on the chip. The optimal pH buffer was determined to be an acetic acid solution with a pH of 5.0 and a concentration of 25 μg/mL. The method for immobilizing the ligand protein on the chip included the following parameters: chip activation for 420s, ligand coupling for 1000s, chip sealing for 420s, and the immobilization amount was 6500RU. The SIL was diluted using a gradient of concentrations ranging from 0.39μM to 100μM. Multi-cycle kinetic operation was then performed with binding for 120s and dissociation for 120s. The original data was imported into Biacore^TM^ Insight Evaluation Software (Version 4.0) and the evaluation of multi-cycle kinetics for samples was selected to calculate kinetic rate constants. Five concentration points were chosen and the kinetic curve was fitted with a 1:1 binding model to obtain the binding rate constant (Ka), dissociation constant (Kd), and affinity constant (KD).

**Cellular thermal shift assay (CETSA)**

HASMCs were treated with or without SIL (10 μM) for 3 hours. For the CETSA experiments ^[30]^, HASMCs were harvested and washed once with PBS. Subsequently, a protease inhibitor cocktail (Roche) was added to the HASMCs and freeze-thawed three times using liquid nitrogen to obtain a cell lysate. Followed by centrifuging at 20,000 g for 20 minutes at 4°C to remove cellular debris. The supernatant was then diluted in PBS and divided into two subgroups: one subgroup was incubated with SIL (10 μM), while the other subgroup was incubated with an empty vehicle. Following a 30-minute incubation at room temperature, the lysates were aliquoted (50 μL per aliquot) and each aliquot was heated to the specified temperatures for 3 minutes, followed by cooling for 3 minutes at room temperature. The heated lysates were then centrifuged at 20,000g for 20 minutes at 4°C to collect the supernatants. The samples were subsequently analyzed by Western blotting.

**Immunofluorescence staining**

HASMCs seeded on cell slides were fixed in 4% paraformaldehyde for 15 minutes and then permeabilized with phosphate-buffered saline (PBS) containing 0.3% Triton X-100 for another 15 minutes. Following blocking with 10% goat serum at room temperature for 30 minutes, the slides were incubated with primary antibodies overnight at 4°C. For aorta tissue staining, 5 μm serial paraffin-embedded sections were dewaxed and underwent antigen retrieval. They were then permeabilized with PBS containing 0.3% Triton X-100 for 15 minutes. The sections were blocked with 10% goat serum at room temperature for 30 minutes and incubated with primary antibodies overnight at 4°C. On a subsequent day, the cell slides and aortic sections were incubated with fluorochrome-labeled secondary antibodies which were diluted in PBS containing 0.1% Triton X-100 for 1 hour at room temperature. The nuclei were counterstained with DAPI (Southern Biotech, United States). The following primary antibodies were used at the indicated dilutions: 4-HNE (1:200, Bioss), GPX4 (1:200, Proteintech, 67763-1-Ig), ACSL4 (1:200, Abcam, ab155282), HMOX1 (1:200, Abconal, A1346), NRF2 (1:400, Abcam, ab62352), TFR (1:200, Proteintech, 10084-2-AP).

**Ferrous Iron Assay**

The Ferrous Iron Assay Kit (Solarbio, Beijing) was used to quantify Fe^2+^ according to the manufacturer's instructions. Briefly, aortic tissues were homogenized in lysis buffer to release Fe^2+^. The supernatant was collected by centrifugation (15,000g, 10 min, 4°C) and incubated with working buffer at 37 °C for 5 min. The supernatant was then obtained by incubation with 100 µL of chloroform and centrifugation. The absorbance of the 96 plate was measured at 593 nm. In addition, the protein levels of aortic tissues were measured with a BCA protein assay kit (23227, Thermo Fisher Scientific), and the level of ferrous iron was normalized to the protein concentration.

**Malondialdehyde (MDA) Assay**

The lipid peroxidation MDA assay kit (S0131, Beyotime, China) was used to determine the level of lipid oxidative stress in aortic tissue according to the manufacturer's instructions. Briefly, human and mouse aortic tissues were harvested, lysed, and centrifuged, and the supernatant was collected for the MDA assay, and a BCA protein assay (23227, Thermo Fisher Scientiﬁc) was used for total protein quantification. The concentration of MDA was calculated from the standard curve and normalized to the protein concentration.

**Supplementary Figure and Table**

**Supplementary Figure S
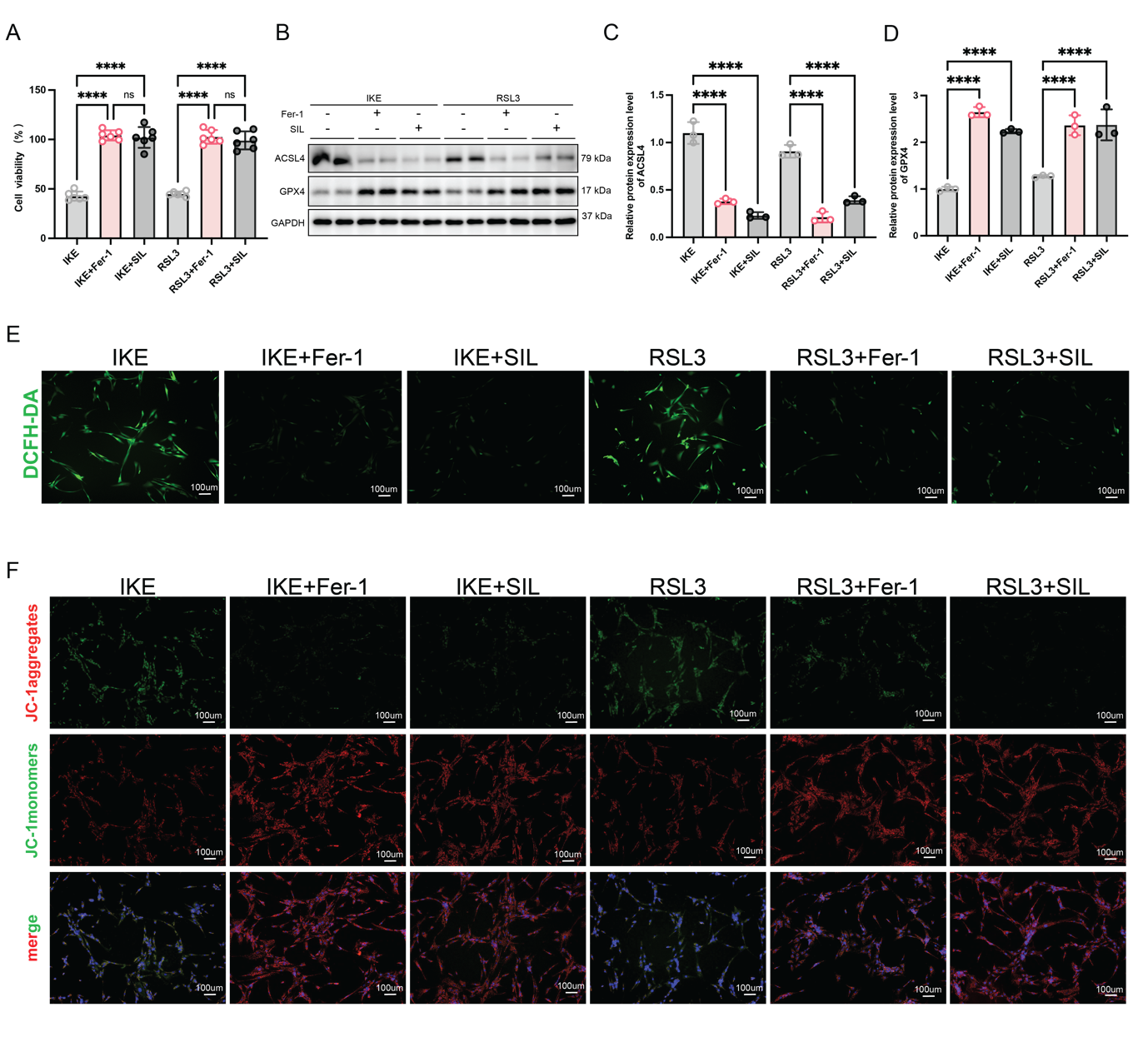
1** Pretreatment with SIL (10 μM) for 24 hours or Fer-1 (2 μM) for 2 hours before IKE exposure (2.5 μM) for 18 hours and RSL3 exposure (50 nM) for 12 hours individually. (A) CCK8 assays were used to assay viability of HASMCs, n=6. (B) Western blot analysis for protein expression of GPX4 and ACSL4 in HASMCs. (C-D) Relative grey value quantitative analysis of GPX4 and ACSL4, n=3. (E) DCFH-DA staining was used for the detection of ROS. Scale bar = 100 μm. (F) JC-1 staining was used to assay the mitochondria membrane potential. Scale bar = 100 μm. Data are expressed as mean ± SD. *****p* < 0.0001; ns: no significant difference.

**
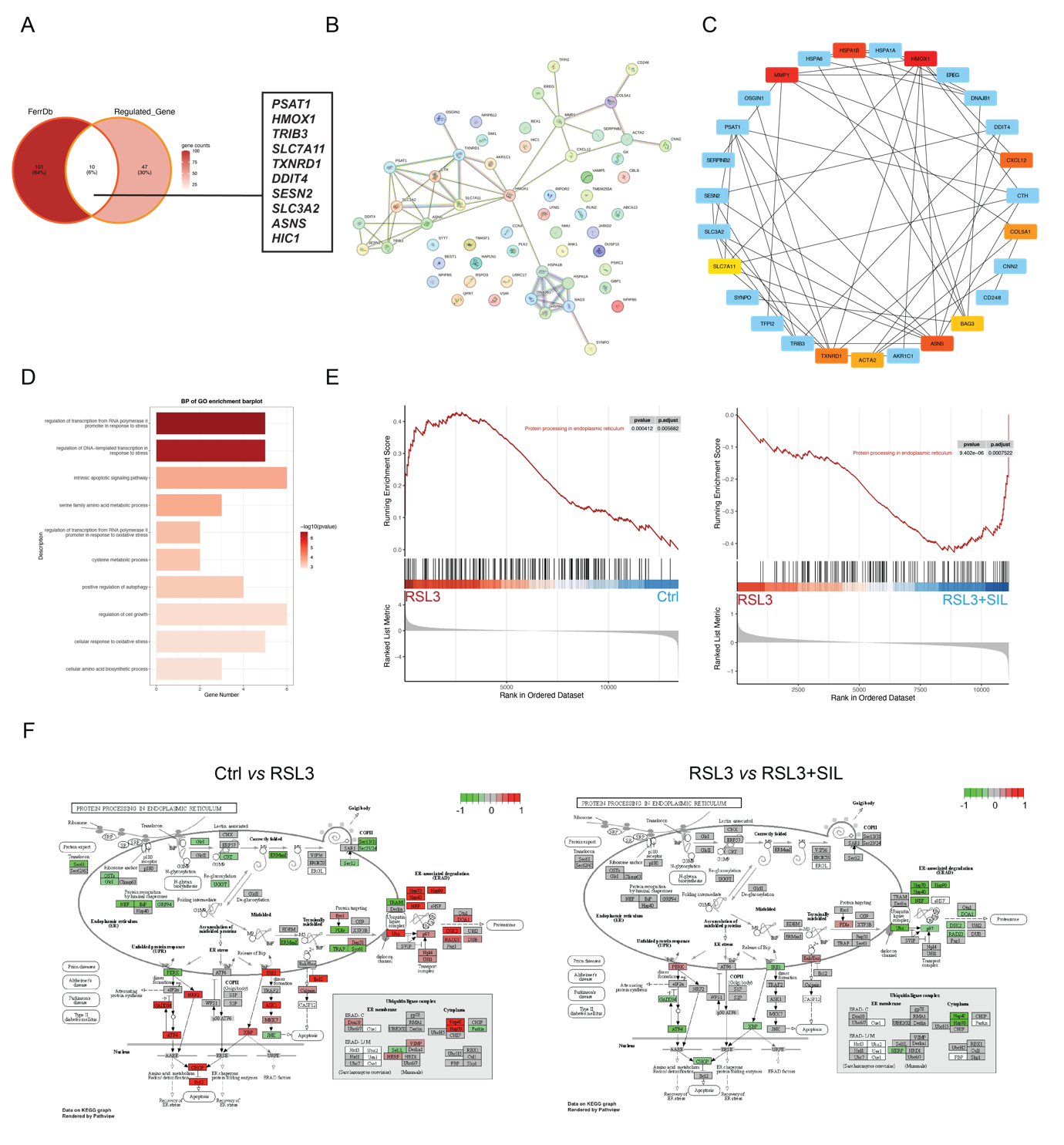
Supplementary Figure S2** (A) Venn diagram illustrating the genes that are common to the DEGs and the FerrDb database. (B) Protein-protein interaction (PPI) analysis of the DEGs. (C) The hub gene of the DEGs. (D) Functional enrichment of the DEGs in the biological process (BP) category of the Gene Ontology (GO). (E) Gene Set Enrichment Analysis (GSEA) of the DEGs. (F) Pathview analysis of protein processing in the endoplasmic reticulum.

**
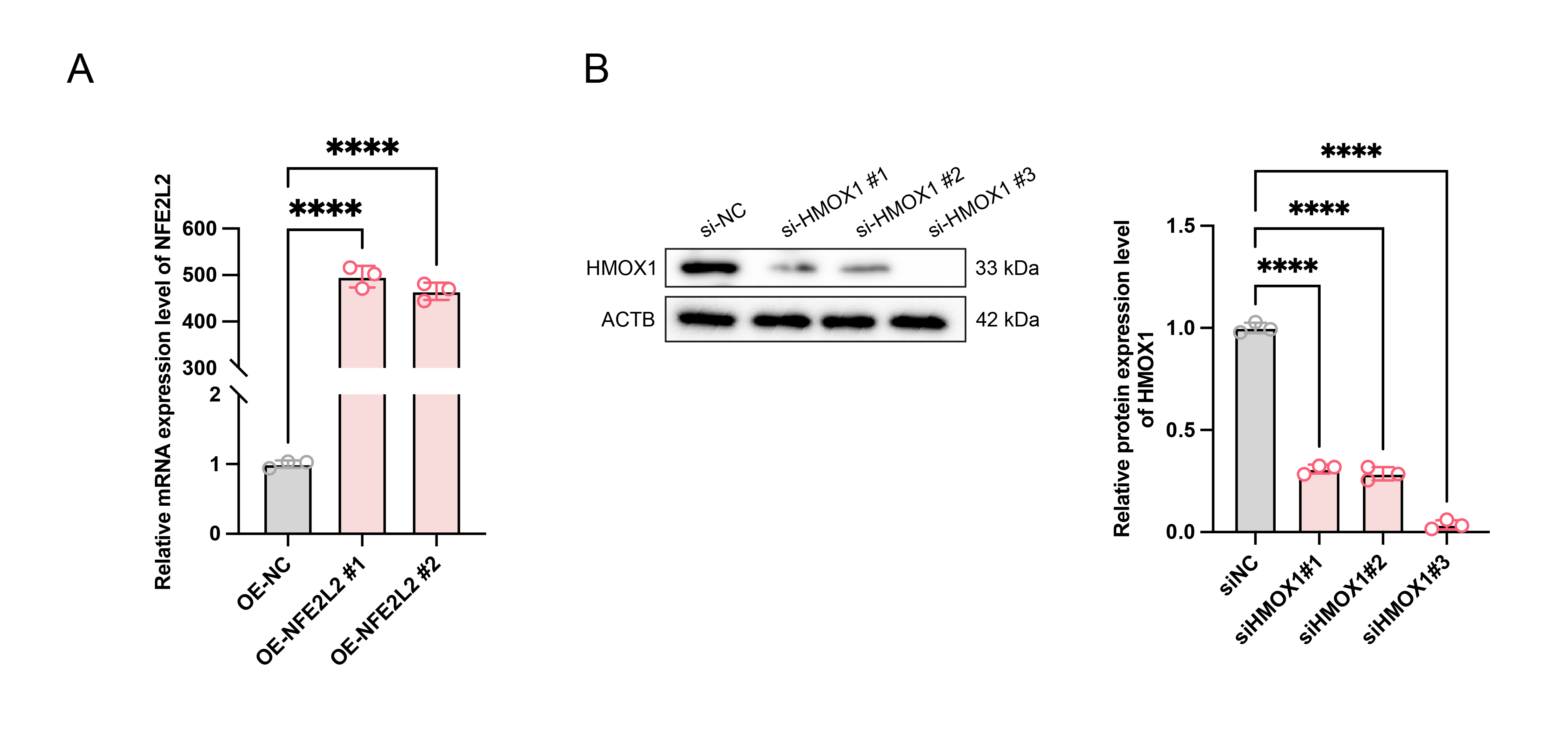
Supplementary Figure S3** (A) qRT-PCR analysis of *NFE2L2*, n=3. (B) Representative immunoblots and densitometric quantification analysis of the protein expression levels of HMOX1, n=3. Data are expressed as mean ± SD. *****p* < 0.0001.

**Supplemental Table S1**

Demographics and Clinical Characteristics

| Characteristics | Controls (N=6) | AD patients (N=10) | *p*-value |
| --- | --- | --- | --- |
| Age, mean ± SD, y | 44.2±7.8 | 52.7±14.3 | 0.146 |
| Gender, n(%) |  |  | 0.607 |
| Female | 3(50.0) | 3(30.0) |  |
| Male | 3(50.0) | 7(70.0) |  |
| Medical history and risk factors, n(%) |  |  |  |
| Smoking | 1(16.7) | 6(60.0) | 0.145 |
| Alcohol | 1(16.7) | 2(20.0) | 1.000 |
| Diabetes mellitus | 2(33.3) | 0(0) | 0.125 |
| Hypertension | 5(83.3) | 7(70.0) | 1.000 |
| Biochemical and  hematological data, mean ± SD |  |  |  |
| White blood cells (×10^9/L) | 7.36±2.15 | 12.02±4.63 | 0.038 |
| Neutrophils (×10^9/L) | 4.74±1.74 | 10.62±4.66 | 0.011 |
| C-reactive protein (mg/L) | 3.04(1.81,30.06) | 18.19(5.74,148.68) | 0.042 |
| Creatinine (μmol/L) | 80.25(48.90,121.10) | 83.50(73.00,135.00) | 0.412 |
| Blood urea nitrogen (mmol/L) | 5.81(4.50,7.55) | 6.55(5.95,9.31) | 0.220 |
| Cholesterol (mmol/L) | 4.37±0.82 | 3.99±0.58 | 0.294 |
| Triglycerides (mmol/L) | 1.82±0.80 | 1.50±0.92 | 0.495 |
| ALT, U/L | 35.33±17.59 | 40.32±23.08 | 0.657 |
| AST, U/L | 28.08±14.52 | 36.36±22.03 | 0.429 |
| Glucose, mmol/L | 6.09±1.32 | 6.86±1.27 | 0.264 |
| D-dimer, ng/ml | 210.0(175.0,475.0) | 2935.0(1532.5,5725.0) | 0.001 |
| Vital signs |  |  |  |
| Heart rate | 86±8 | 90±16 | 0.598 |
| Systolic pressure(mmHg) | 123.7±6.0 | 168.1±40.5 | 0.007 |
| Diastolic pressure(mmHg) | 78.5±10.6 | 91.6±18.6 | 0.139 |
| CTA image data |  |  |  |
| Aortic diameter（mm） | 30.2±5.9 | 49.3±7.9 | 0.000 |

**Supplemental Table S2**

The sequences of interfering RNA (siRNA)

| **Gene** | **Sequences (5′-3′)** |
| --- | --- |
| Human-*HMOX1*-siRNA#1 | sense: GCUGAGUUCAUGAGGAACUUUTT  antisense: AAAGUUCCUCAUGAACUCAGCTT |
| Human-*HMOX1*-siRNA#2 | sense: ACAGUUGCUGUAGGGCUUUAUTT  antisense: AUAAAGCCCUACAGCAACUGUTT |
| Human-*HMOX1*-siRNA#3 | sense: GCUCAACAUCCAGCUCUUUTT  antisense: AAAGAGCUGGAUGUUGAGCAG |
|  |  |

**Supplemental Table S3** The primer sequences used for quantitative real-time PCR

| **Gene(mRNA)** | **Primer (5′-3′)** |
| --- | --- |
| Human *NFE2L2* | Forward: AGACGGTATGCAACAGGACA  Reverse: ACCATGGTAGTCTCAACCAGC |
| Human *HMOX1* | Forward: TGACCCATGACACCAAGGAC  Reverse: AGTGTAAGGACCCATCGGAGA |
| Human *FTH1* | Forward: AGCTCTACGCCTCCTACGTT  Reverse: CCTGAAGGAAGATTCGGCCA |
| Human *TFRC* | Forward: GGCTGTATTCTGCTCGTGGA  Reverse: CCCCAGAAGACATGTCGGAAA |
| Human *SLC40A1* | Forward: TACTTGGGGAGATCGGATGTG  Reverse: ATTCTGTACCACCAGCGAGG |
| Human *GAPDH* | Forward: AAGGCTGTGGGCAAGG  Reverse: TGGAGGAGTGGGTGTCG |
