# Supplementary material for: Dual functions of silibinin in attenuating aortic dissection via regulating iron homeostasis and endoplasmic reticulum stress against ferroptosis
Source: Cell Death Dis. 2024 Dec 18;15(12):900. doi: 10.1038/s41419-024-07309-x (PMC11655547; doi:10.1038/s41419-024-07309-x)

Western blots images for peer review

Figure 1D

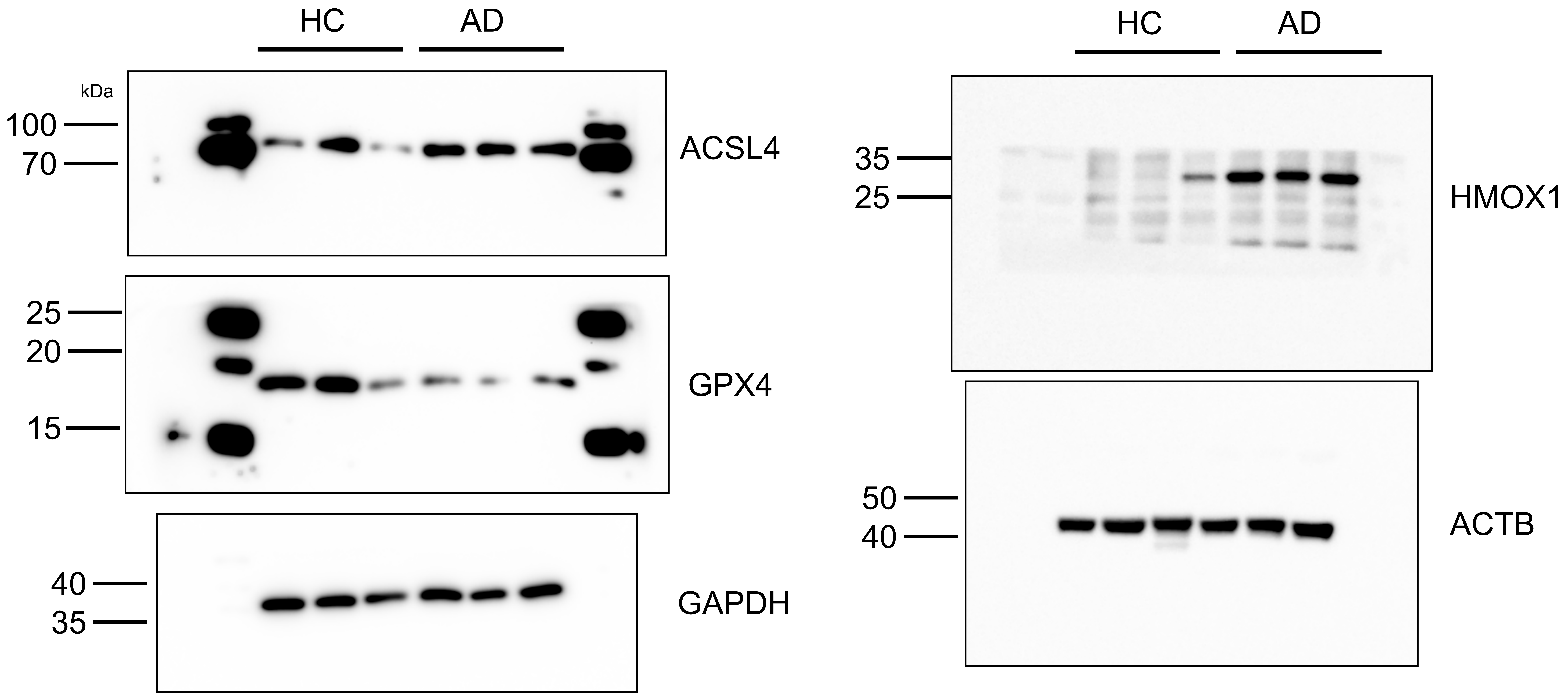

HC
AD
kDa
100
70
ACSL4
25
20
15
GPX4
40
35
GAPDH
HC
AD
35
25
HMOX1
50
40
ACTB

Figure 2G

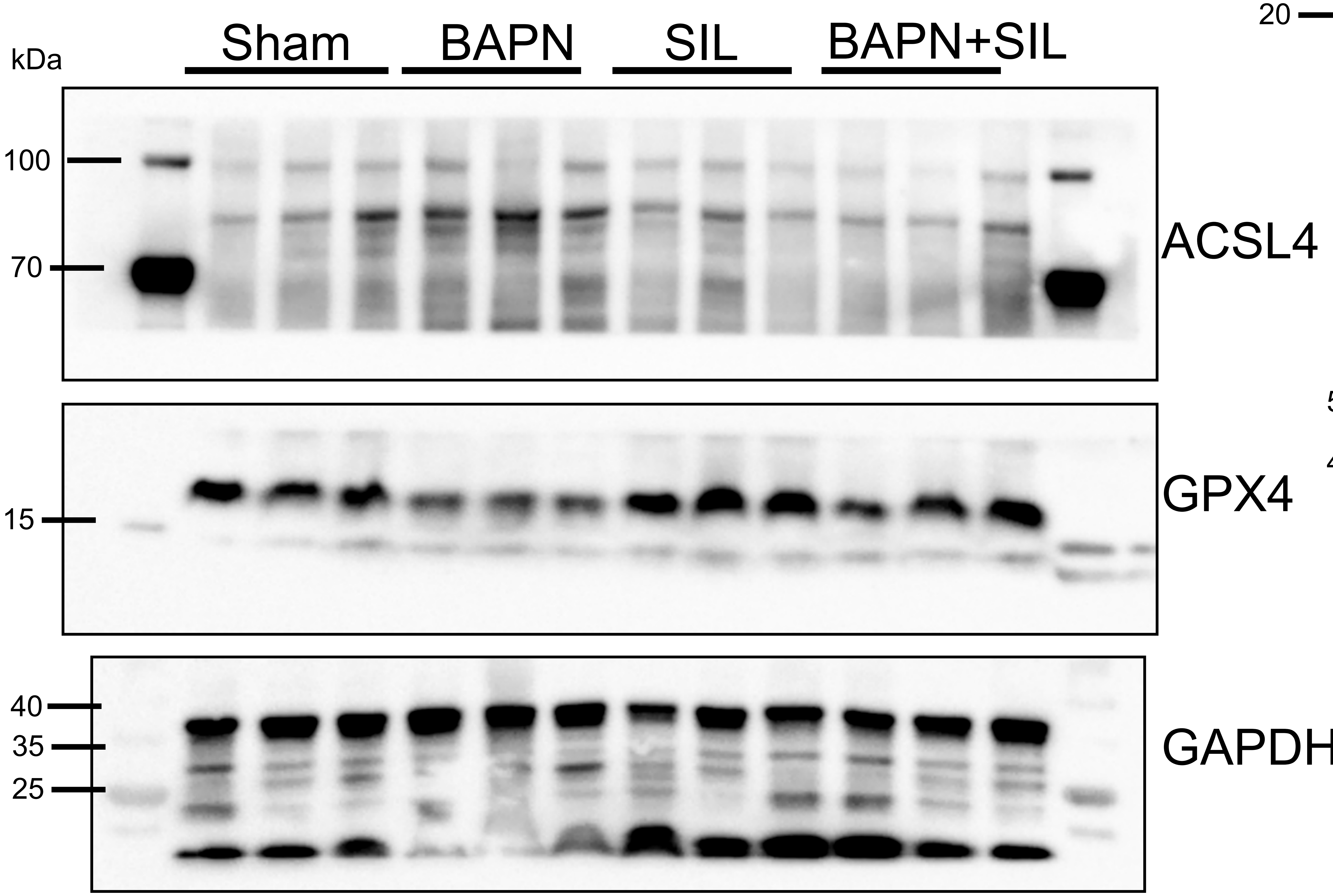

Sham
BAPN
SIL
BAPN+SIL
kDa
100
70
ACSL4
15
GPX4
40
35
25
GAPDH

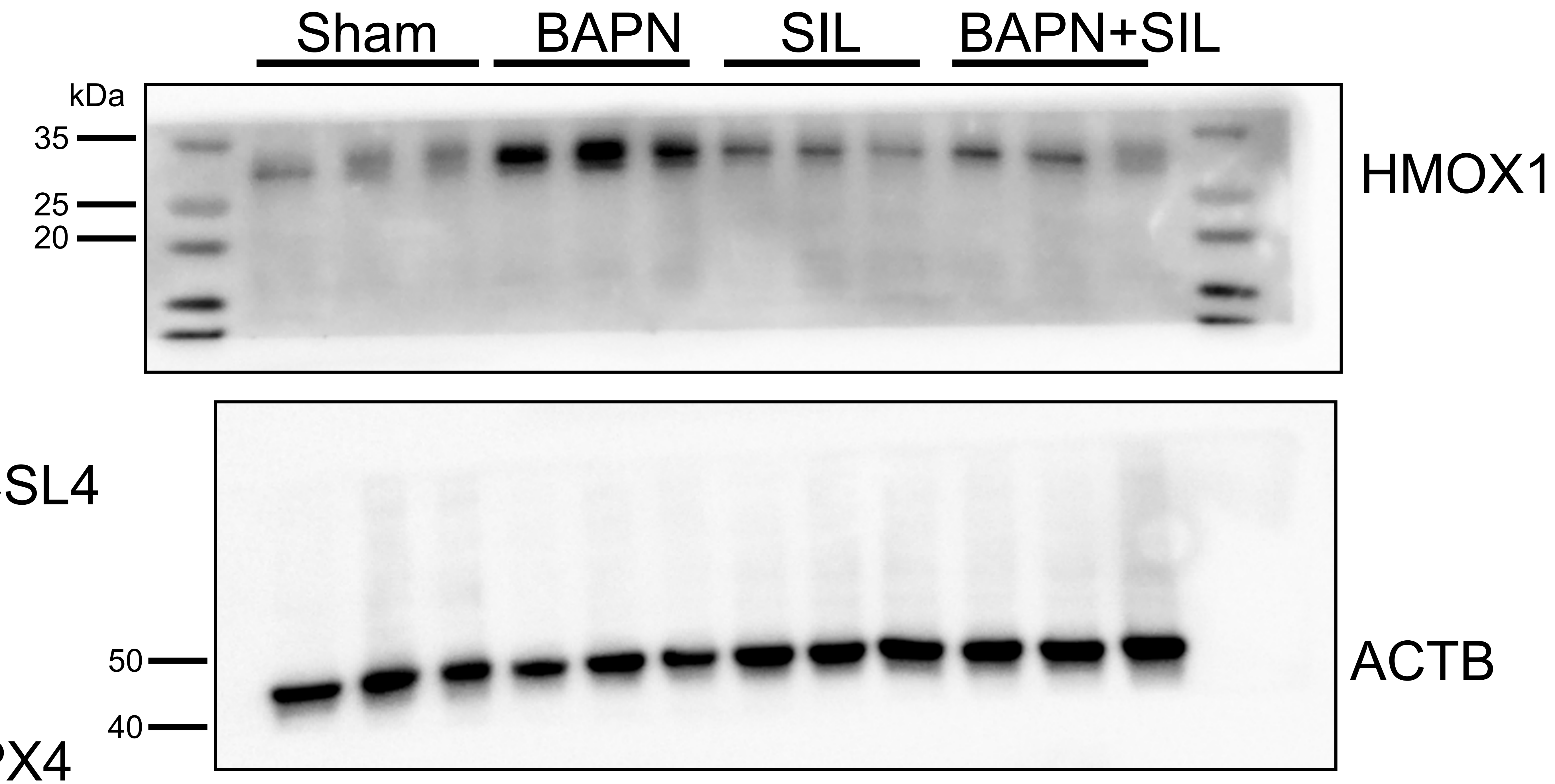

Sham
BAPN
SIL
BAPN+SIL
kDa
35
25
20
HMOX1
50
40
ACTB

Figure 3G

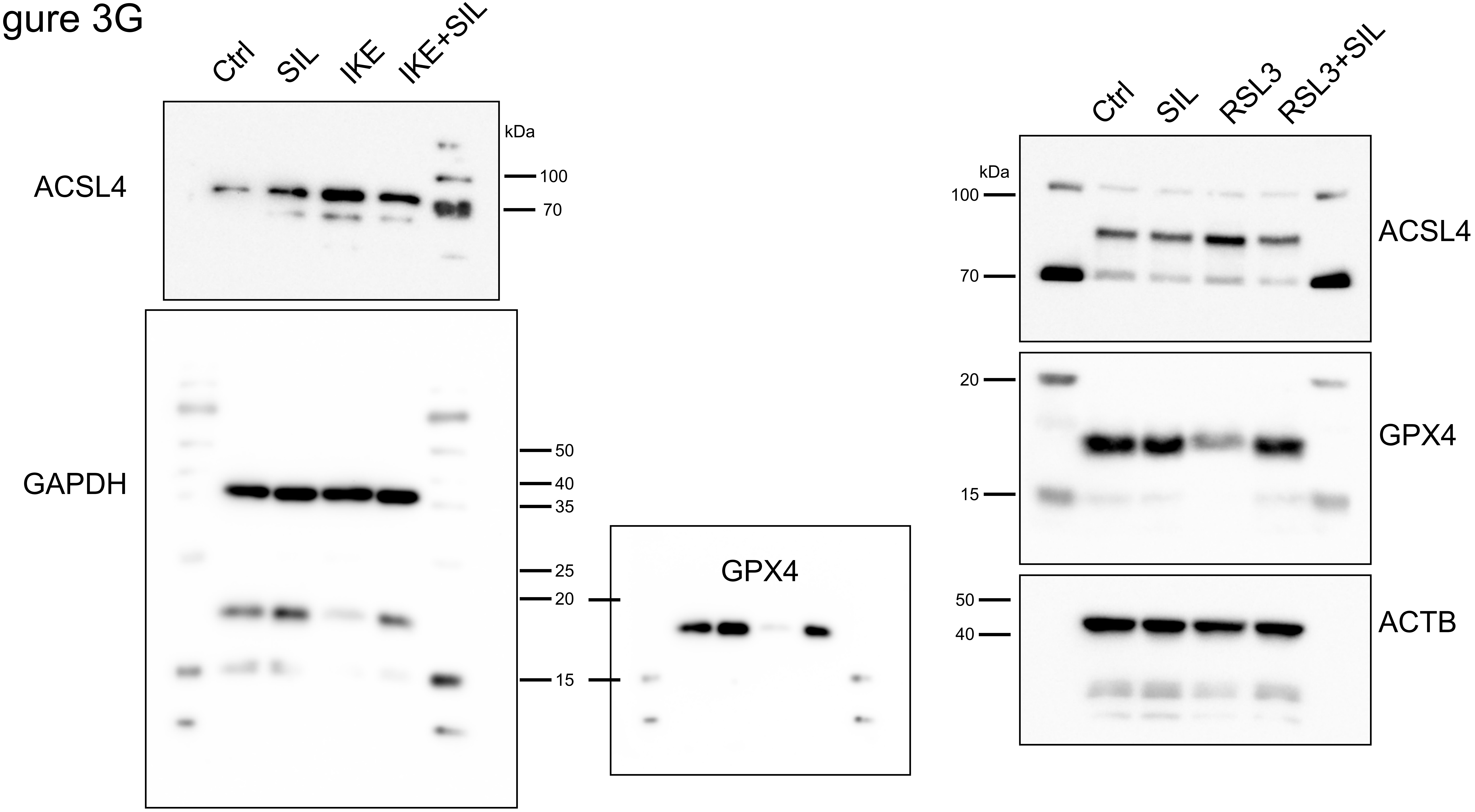
Ctrl
SIL
IKE
IKE+SIL
ACSL4
kDa
100
70
GAPDH
50
40
35
25
20
15
GPX4
Ctrl
SIL
RSL3
RSL3+SIL
kDa
100
70
ACSL4
20
15
GPX4
50
40
ACTB

Figure 5C

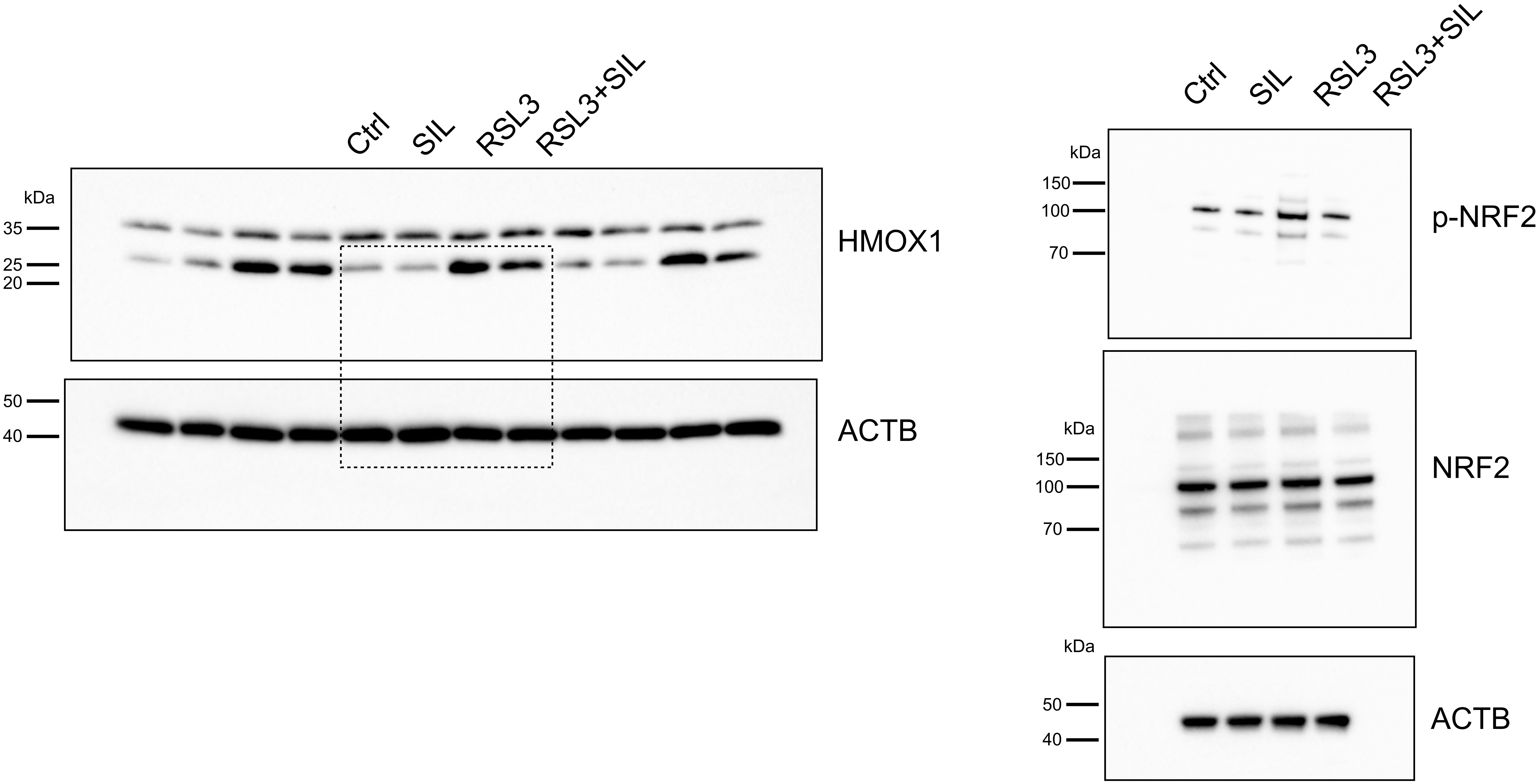

Ctrl
SIL
RSL3
RSL3+SIL
kDa
35
25
20
HMOX1
50
40
ACTB
Ctrl
SIL
RSL3
RSL3+SIL
kDa
150
100
70
p-NRF2
kDa
150
100
70
NRF2
kDa
50
40
ACTB

Figure 5E

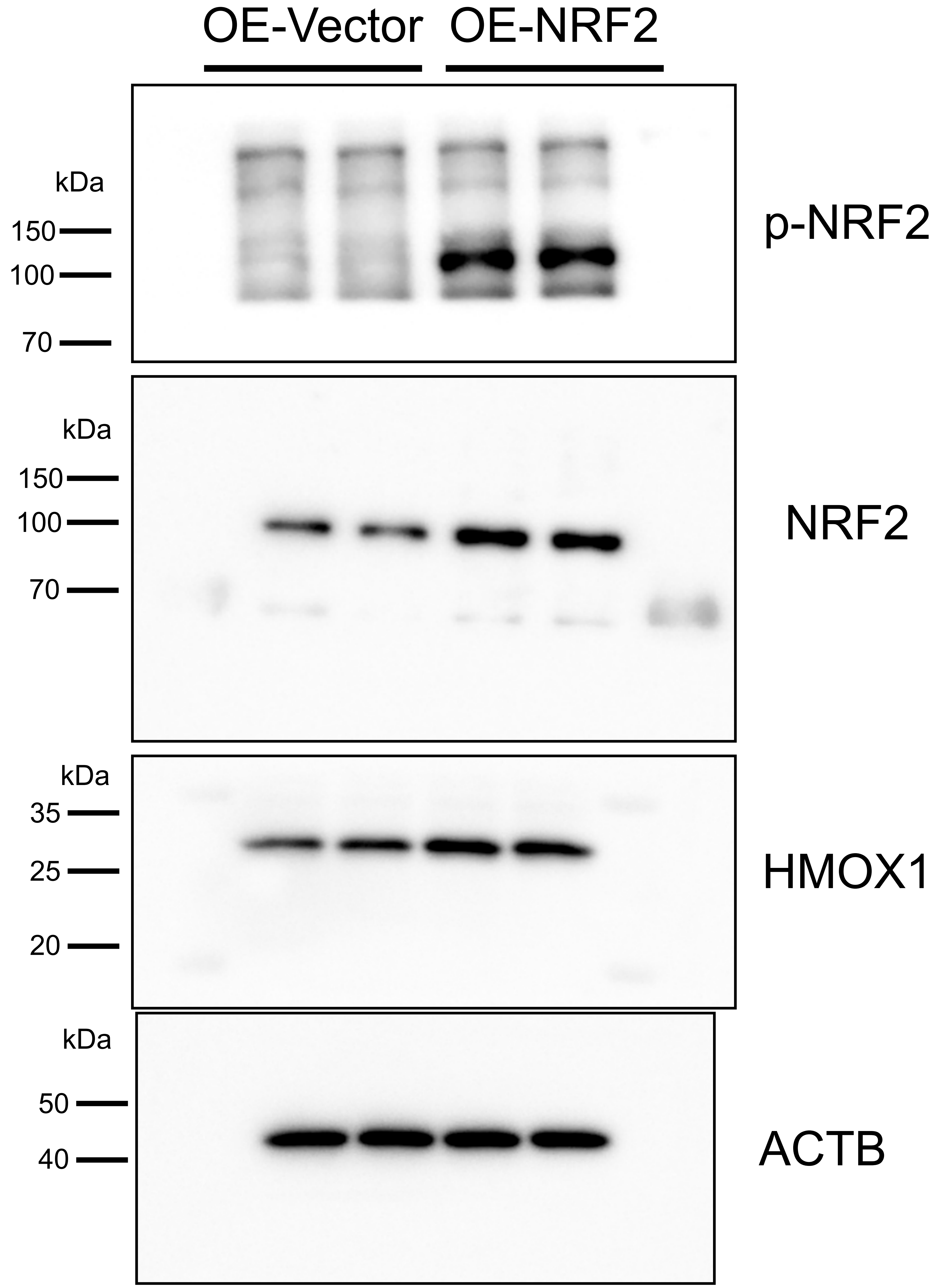

Figure 5G

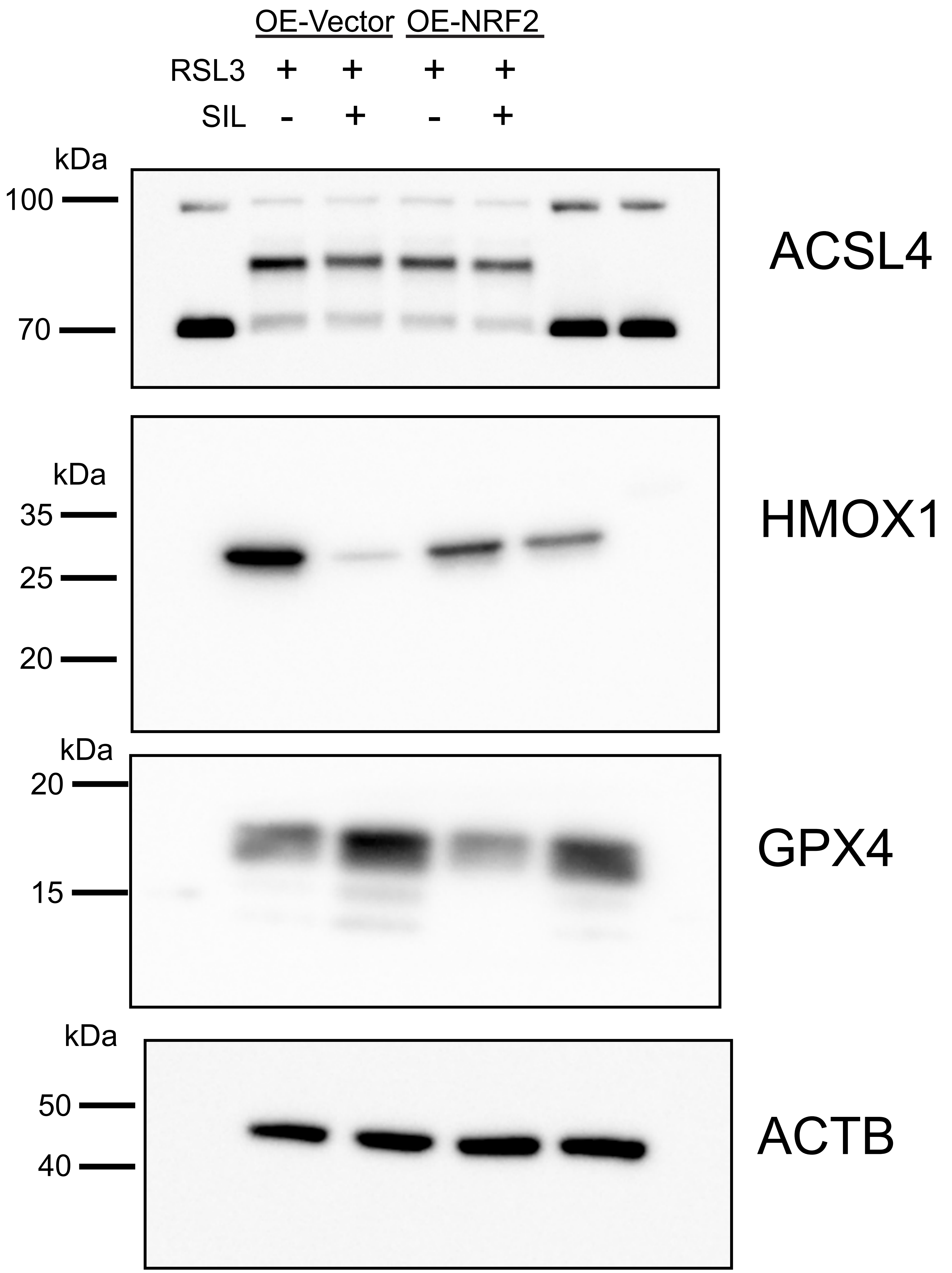

Figure 5L

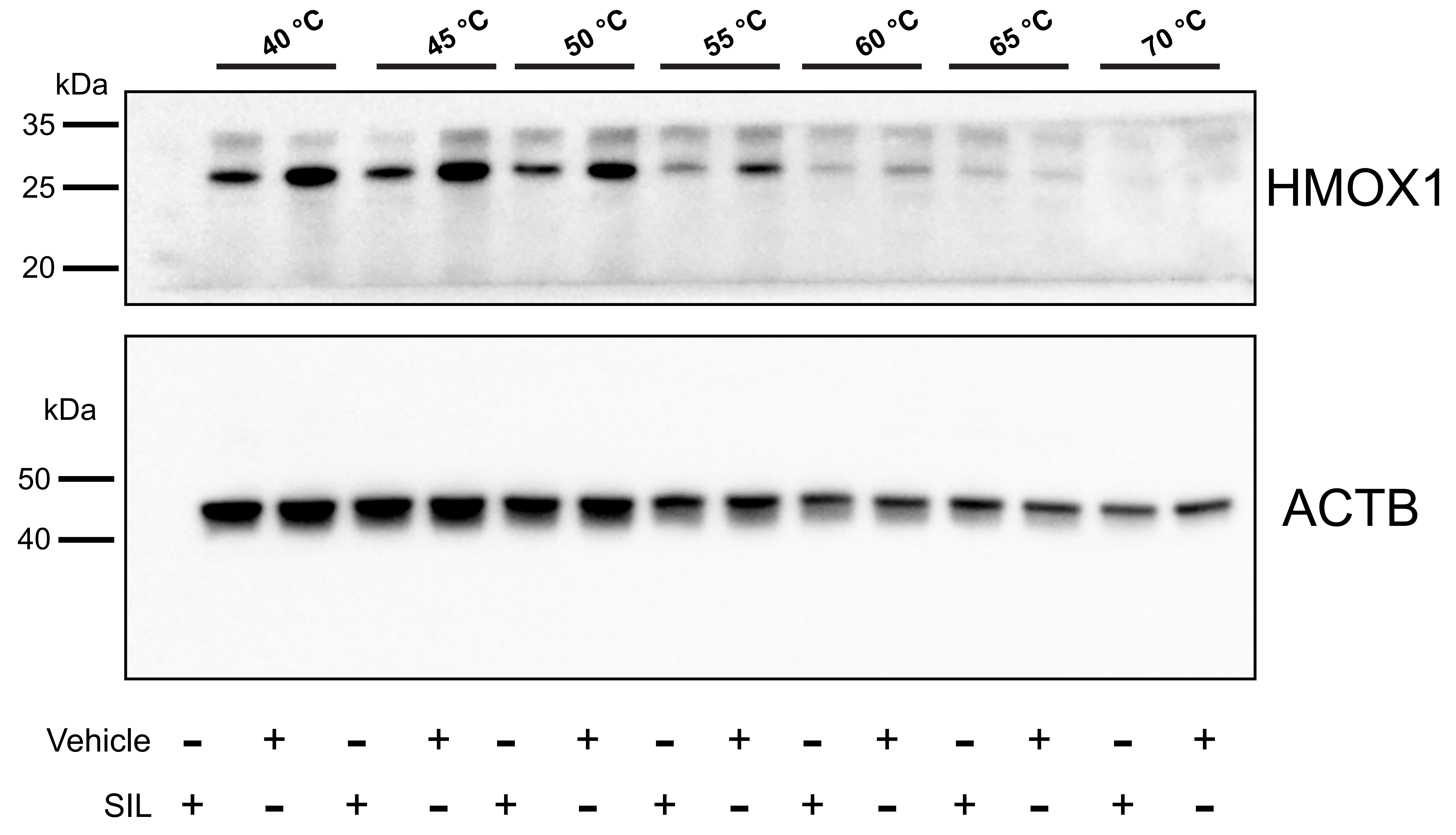

Figure 5M

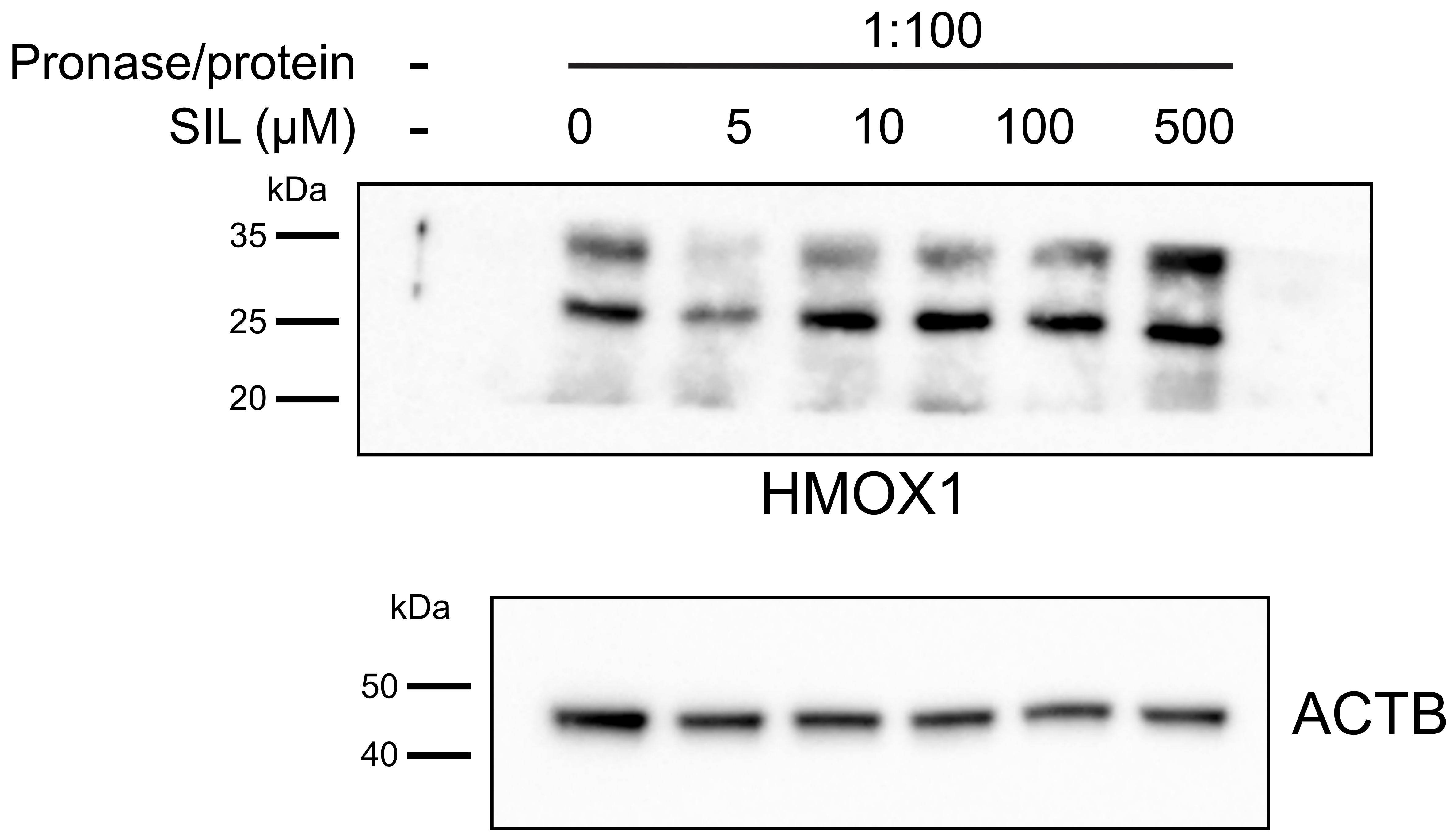

Figure 6B

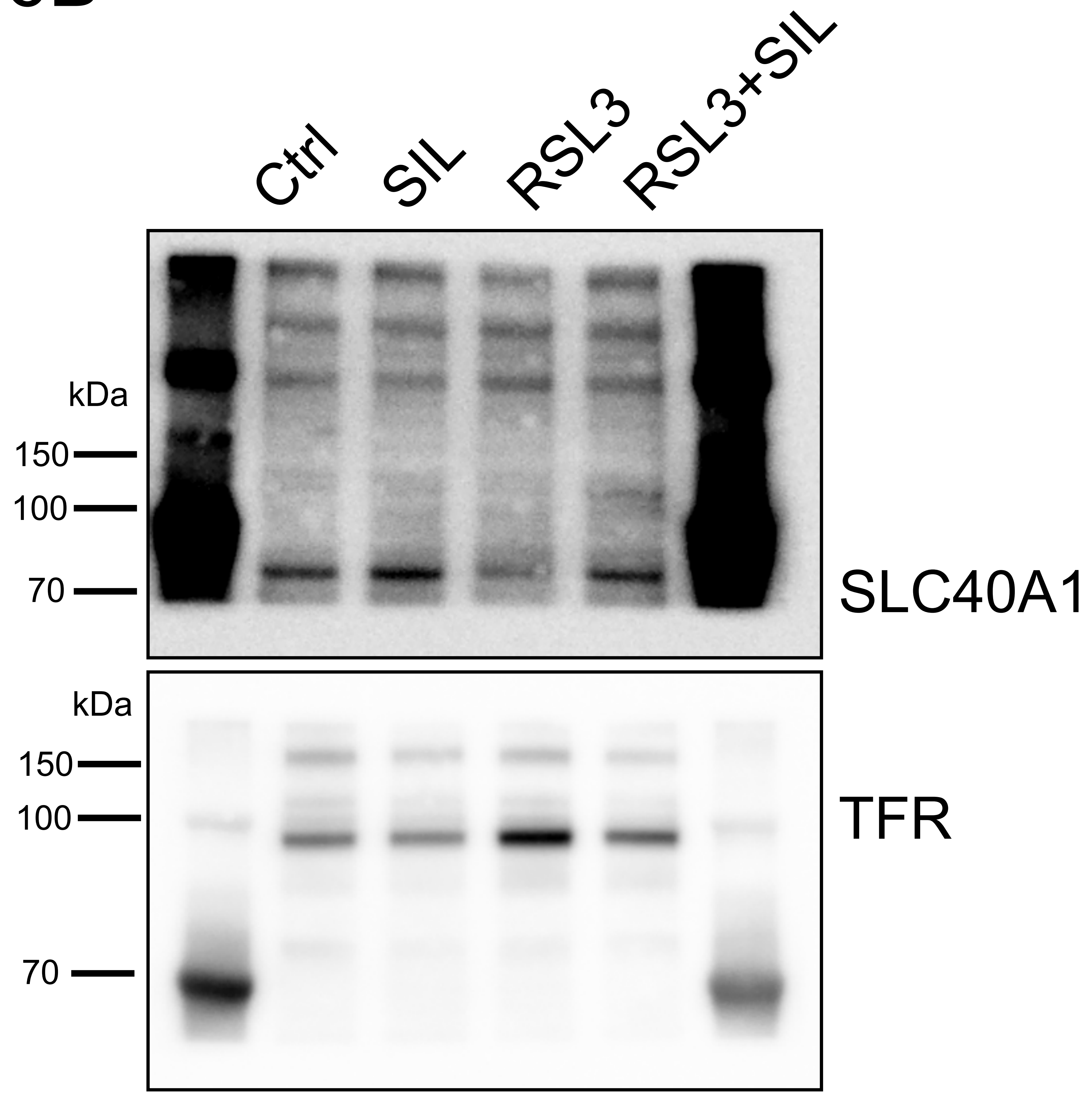

Ctrl
SIL
RSL3
RSL3+SIL
kDa
150
100
70
SLC40A1
kDa
150
100
70
TFR

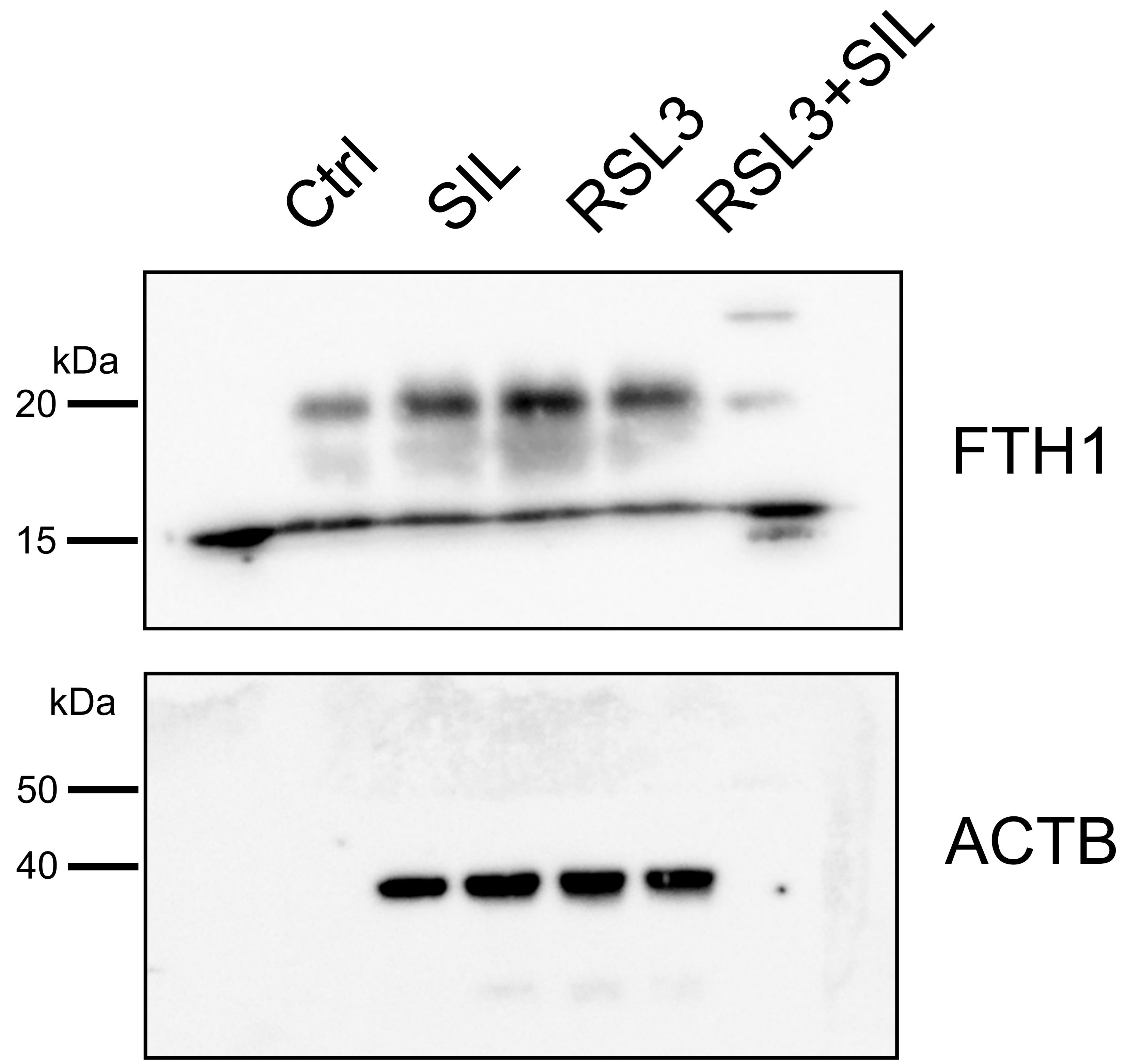

Ctrl
SIL
RSL3
RSL3+SIL
kDa
20
15
FTH1
kDa
50
40
ACTB

Figure 6F

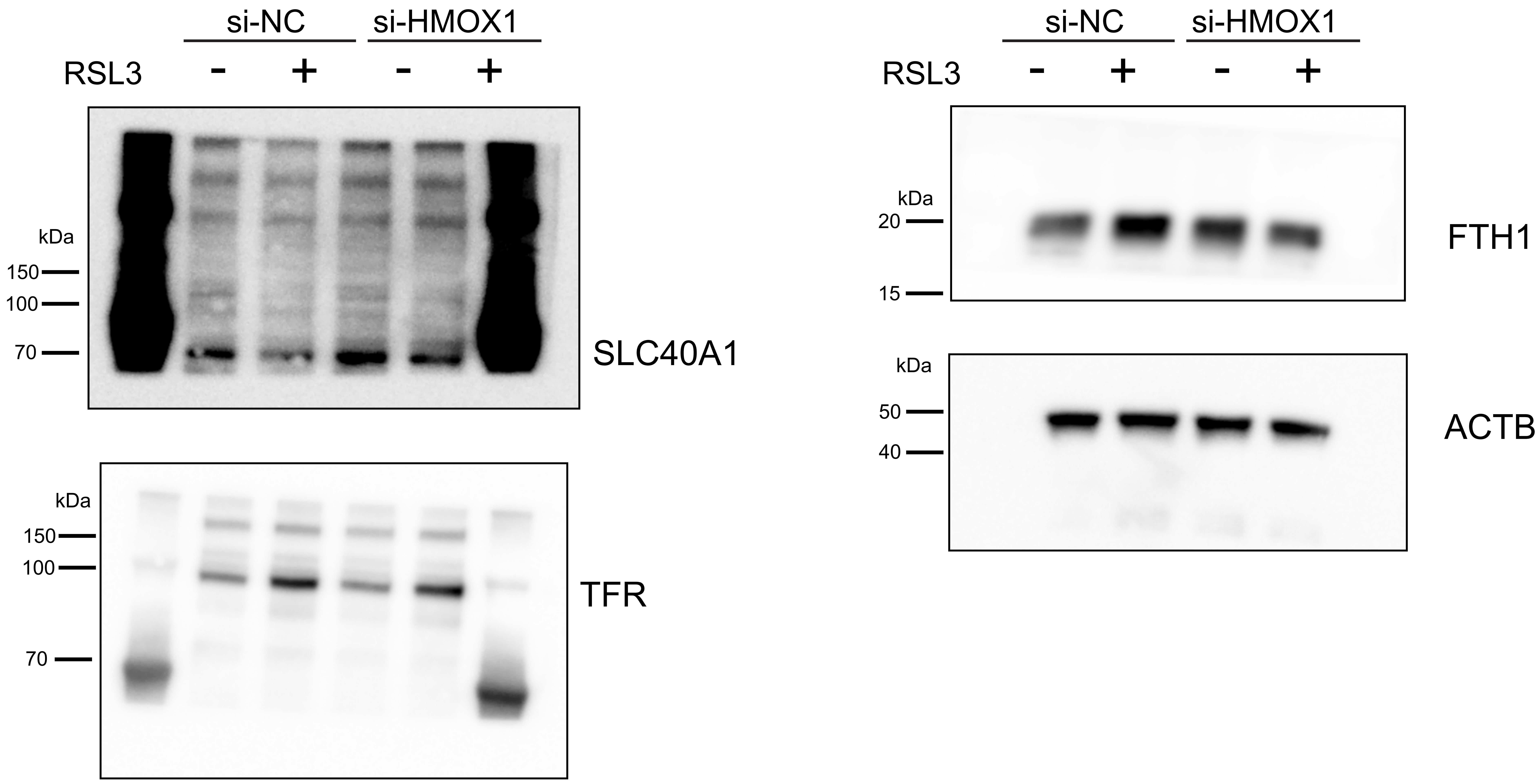
si-NC
si-HMOX1
RSL3
-
+
-
+
kDa
150
100
70
SLC40A1
kDa
150
100
70
TFR
si-NC
si-HMOX1
RSL3
-
+
-
+
kDa
20
15
FTH1
kDa
50
40
ACTB

Figure 6I

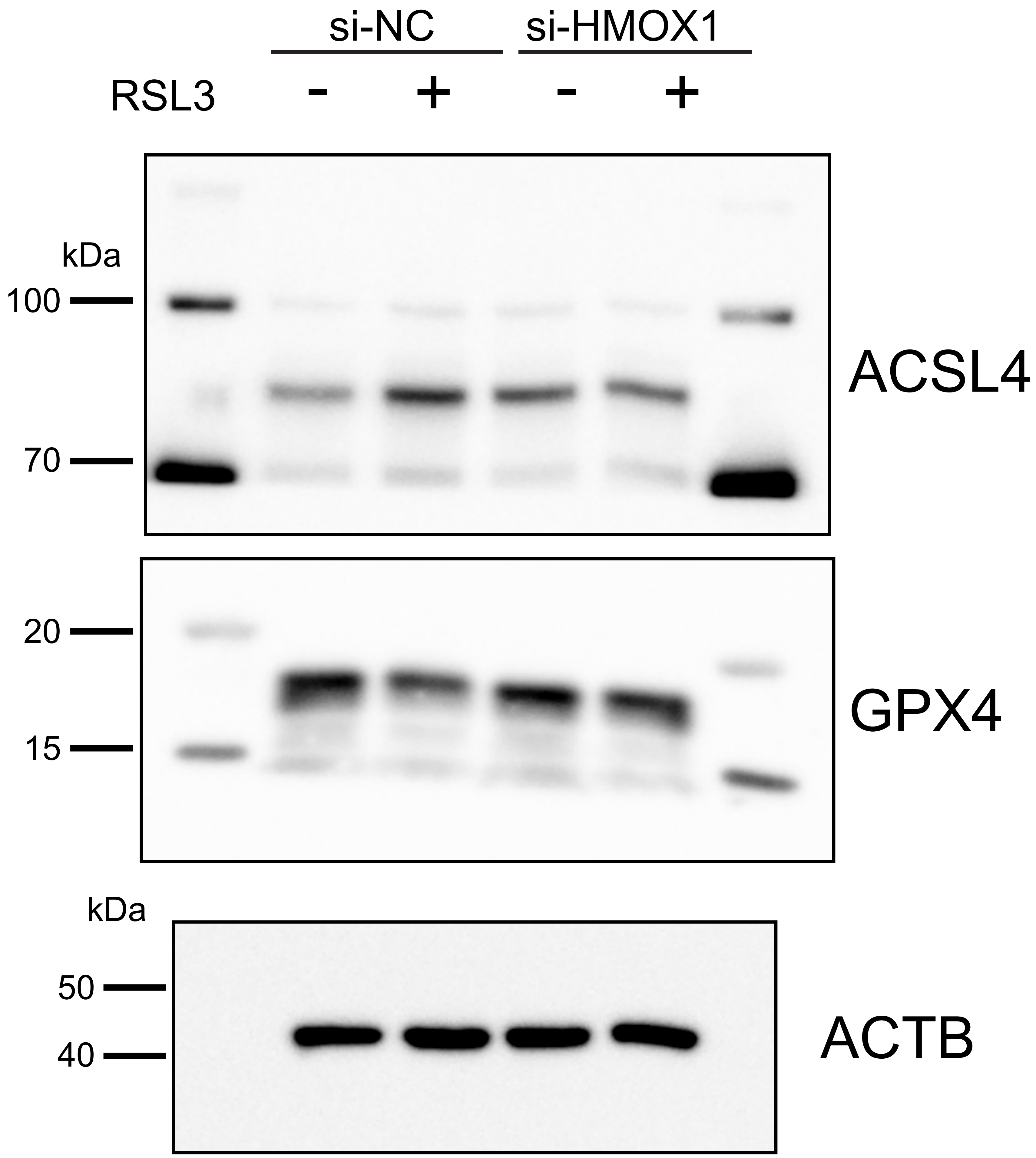
si-NC
si-HMOX1
RSL3
-
+
-
+
kDa
100
70
ACSL4
20
15
GPX4
kDa
50
40
ACTB

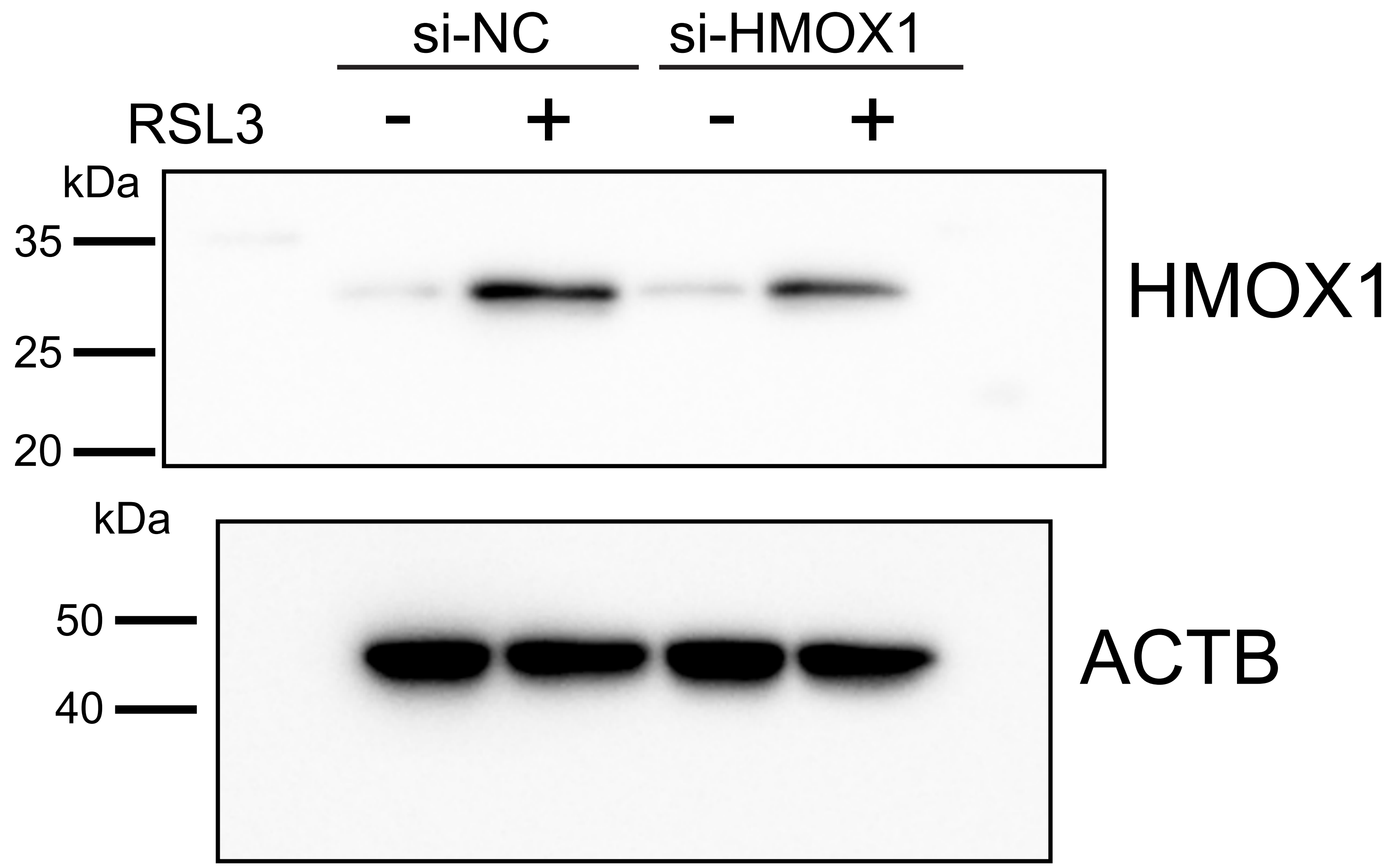
si-NC
si-HMOX1
RSL3
-
+
-
+
kDa
35
25
20
HMOX1
kDa
50
40
ACTB

Figure 7B

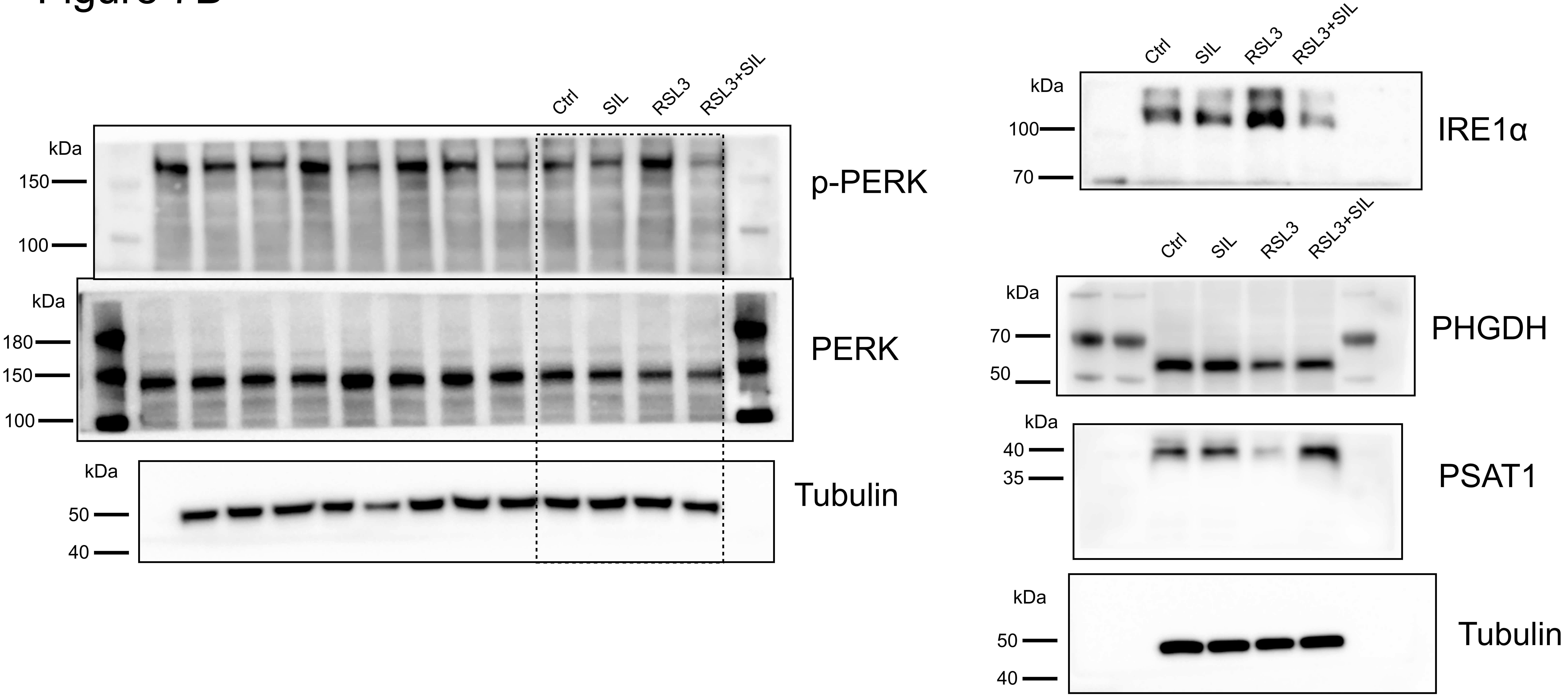

Ctrl
SIL
RSL3
RSL3+SIL
kDa
150
100
p-PERK
kDa
180
150
100
PERK
kDa
50
40
Tubulin
Ctrl
SIL
RSL3
RSL3+SIL
kDa
100
70
IRE1α
Ctrl
SIL
RSL3
RSL3+SIL
kDa
70
50
PHGDH
kDa
40
35
PSAT1
kDa
50
40
Tubulin

Figure 7D

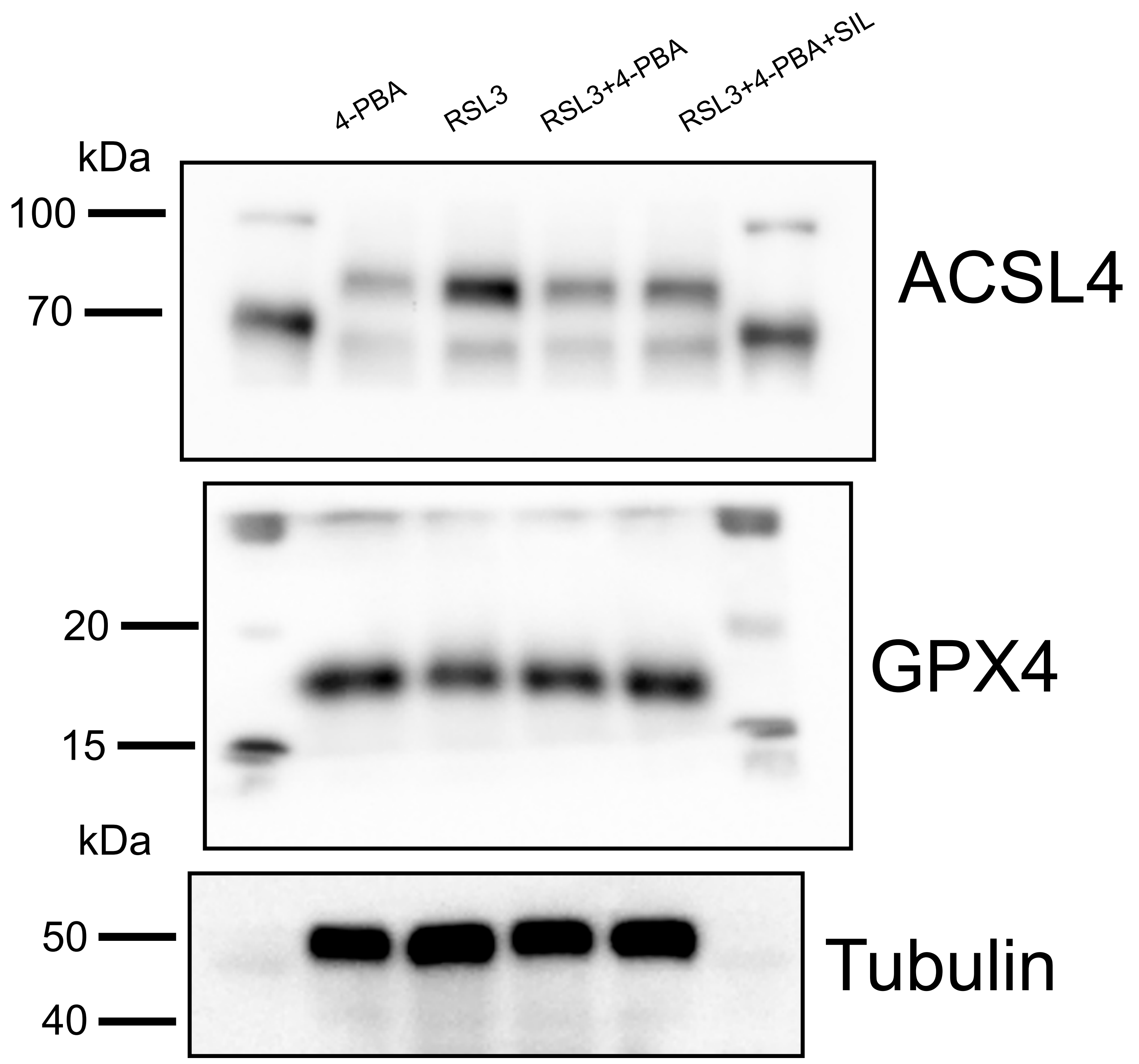

Figure 7F

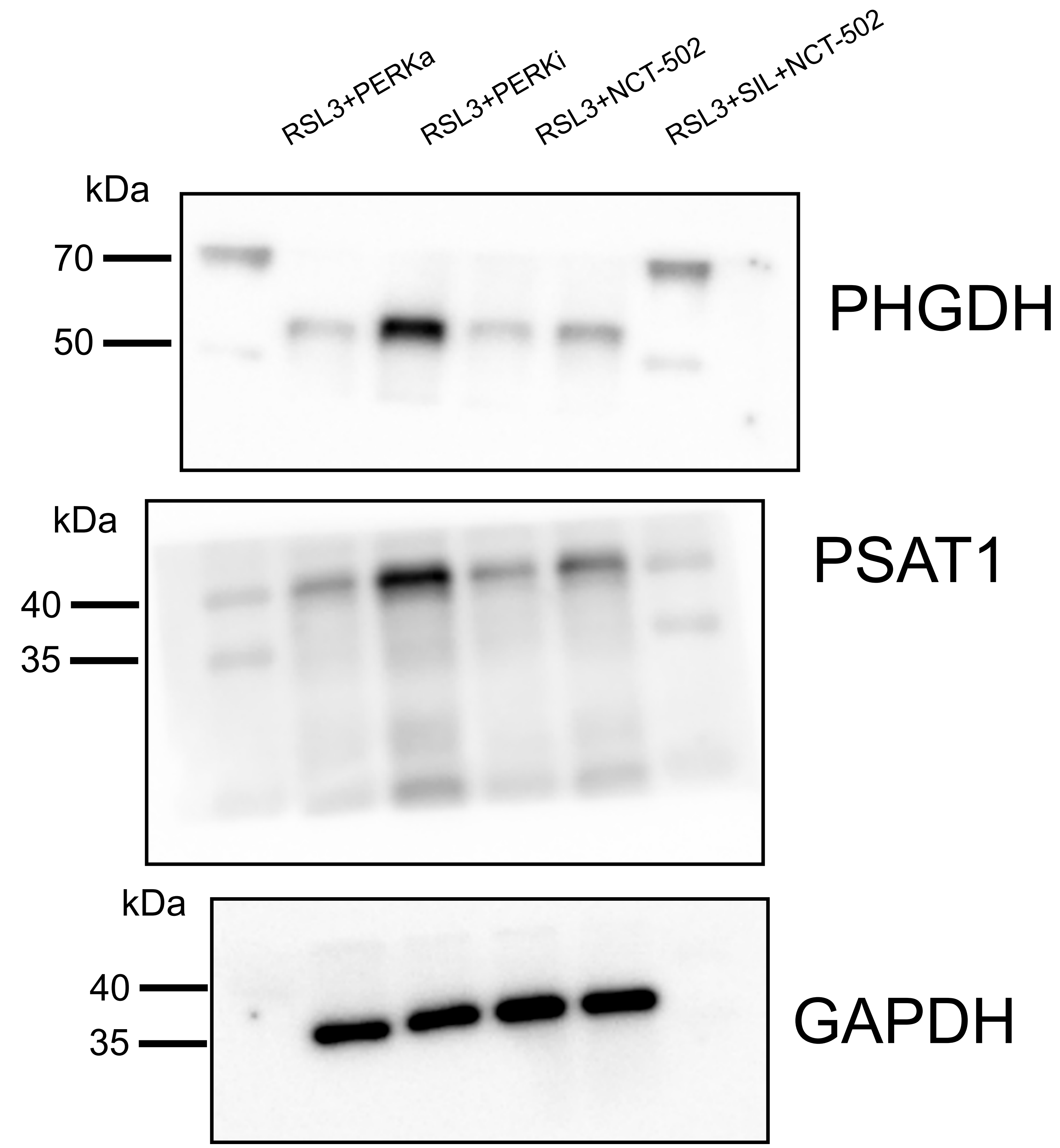

Figure 7H

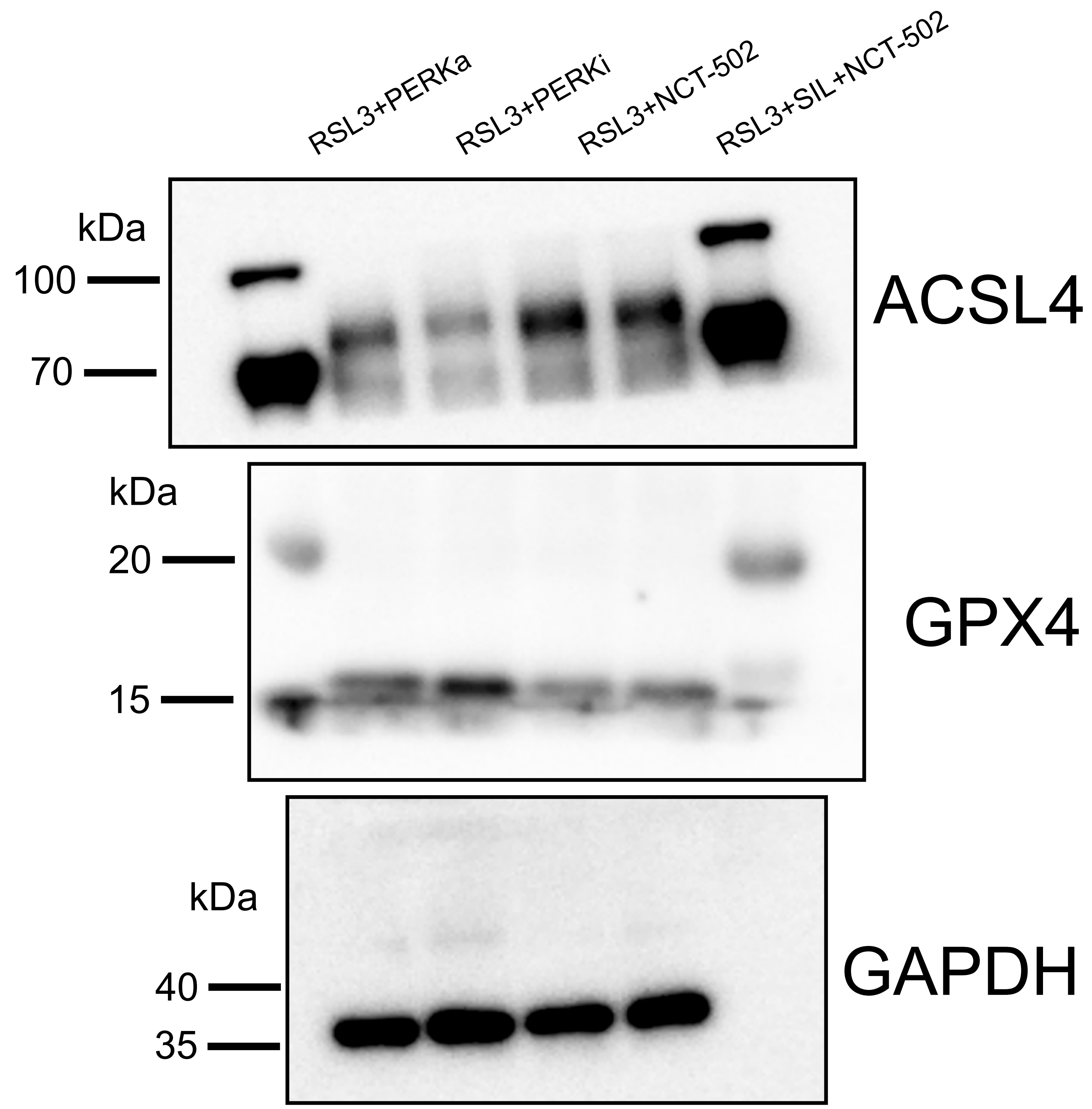

Figure 8B

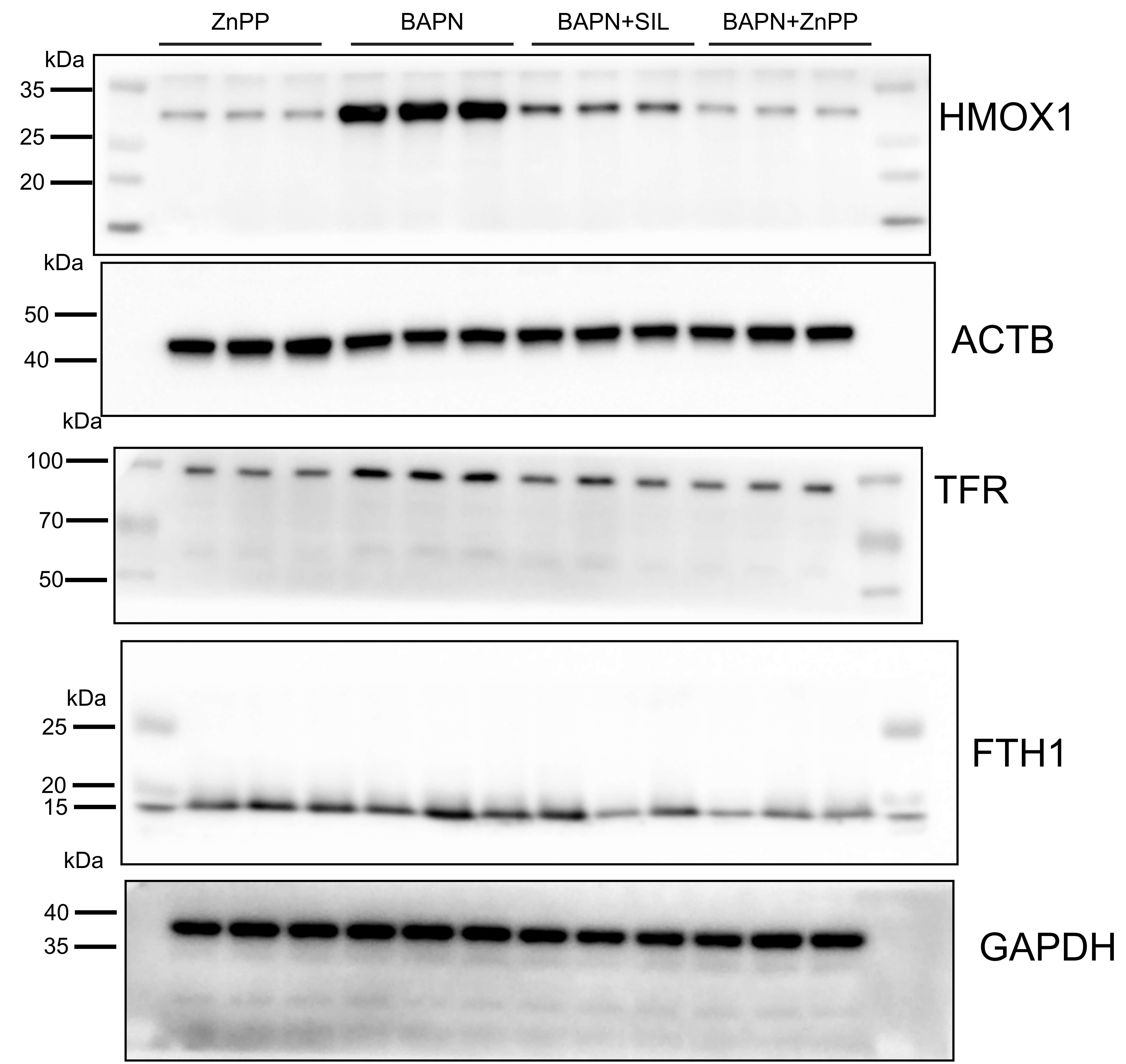

ZnPP
BAPN
BAPN+SIL
BAPN+ZnPP
kDa
35
25
20
HMOX1
kDa
50
40
ACTB
kDa
100
70
50
TFR
kDa
25
20
15
FTH1
kDa
40
35
GAPDH

Figure 8D

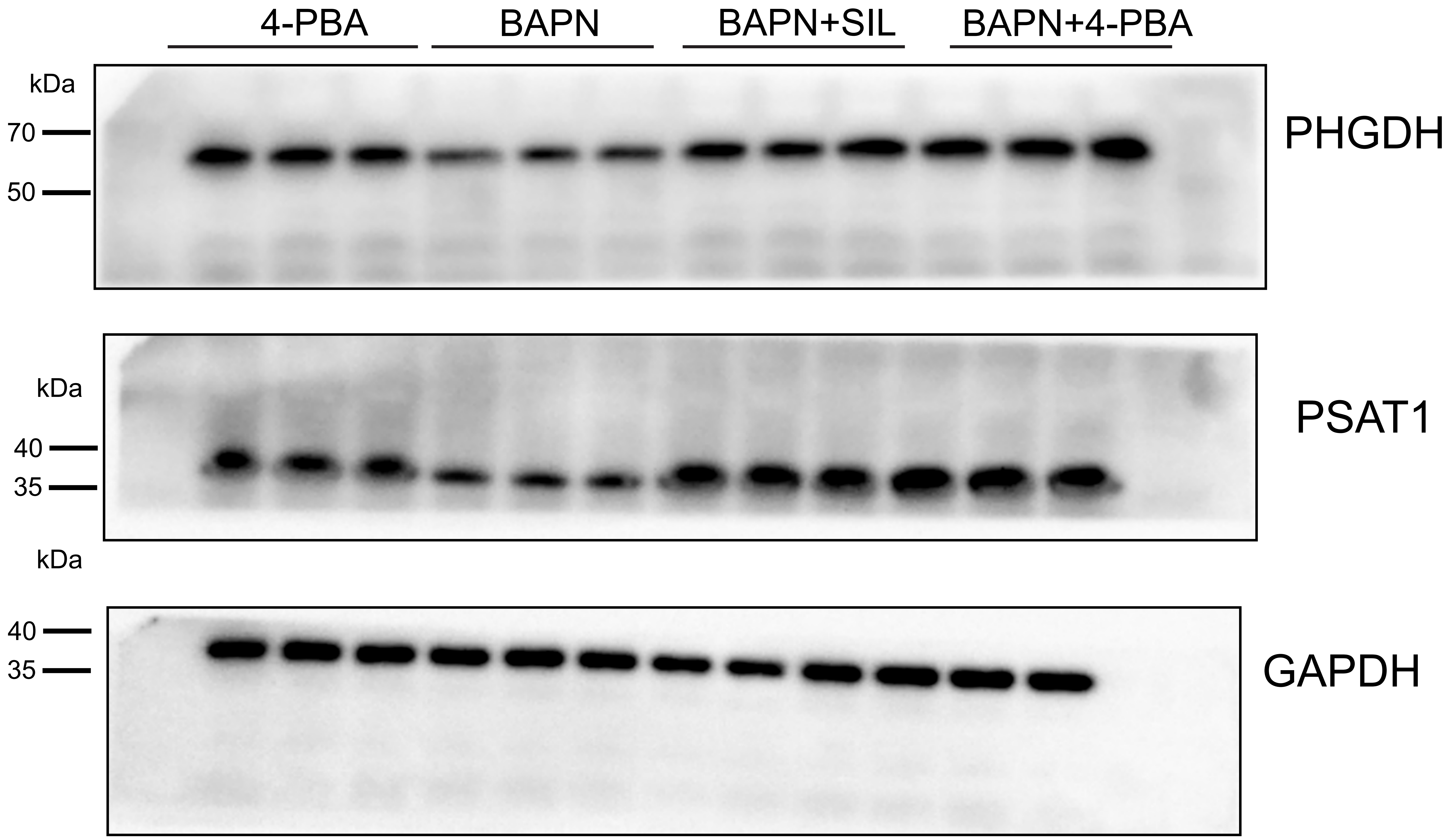
4-PBA
BAPN
BAPN+SIL
BAPN+4-PBA
kDa
70
50
PHGDH
kDa
40
35
PSAT1
kDa
40
35
GAPDH

Figure 8J

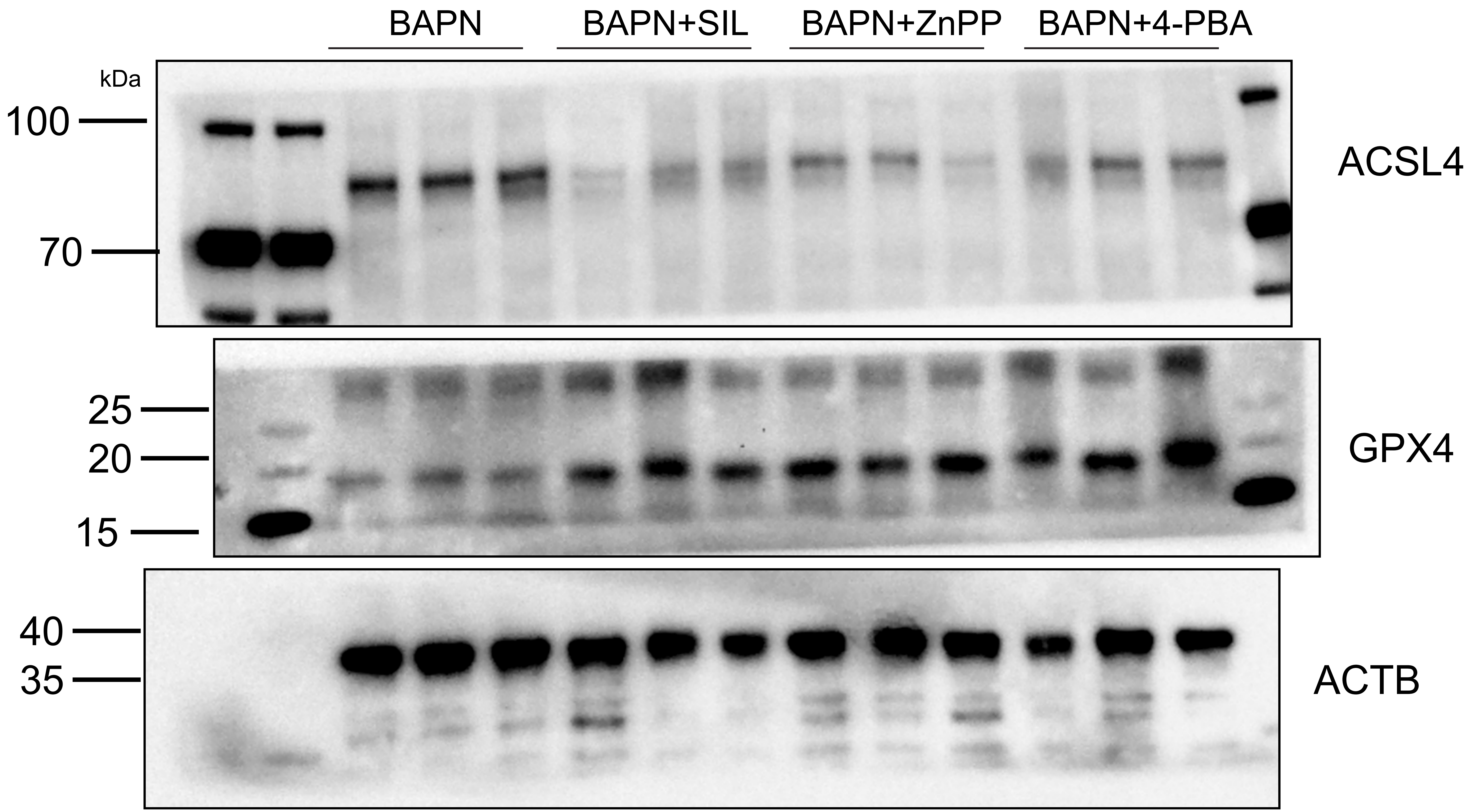
BAPN
BAPN+SIL
BAPN+ZnPP
BAPN+4-PBA
kDa
100
70
ACSL4
25
20
15
GPX4
40
35
ACTB

Supplementary Figure 1B

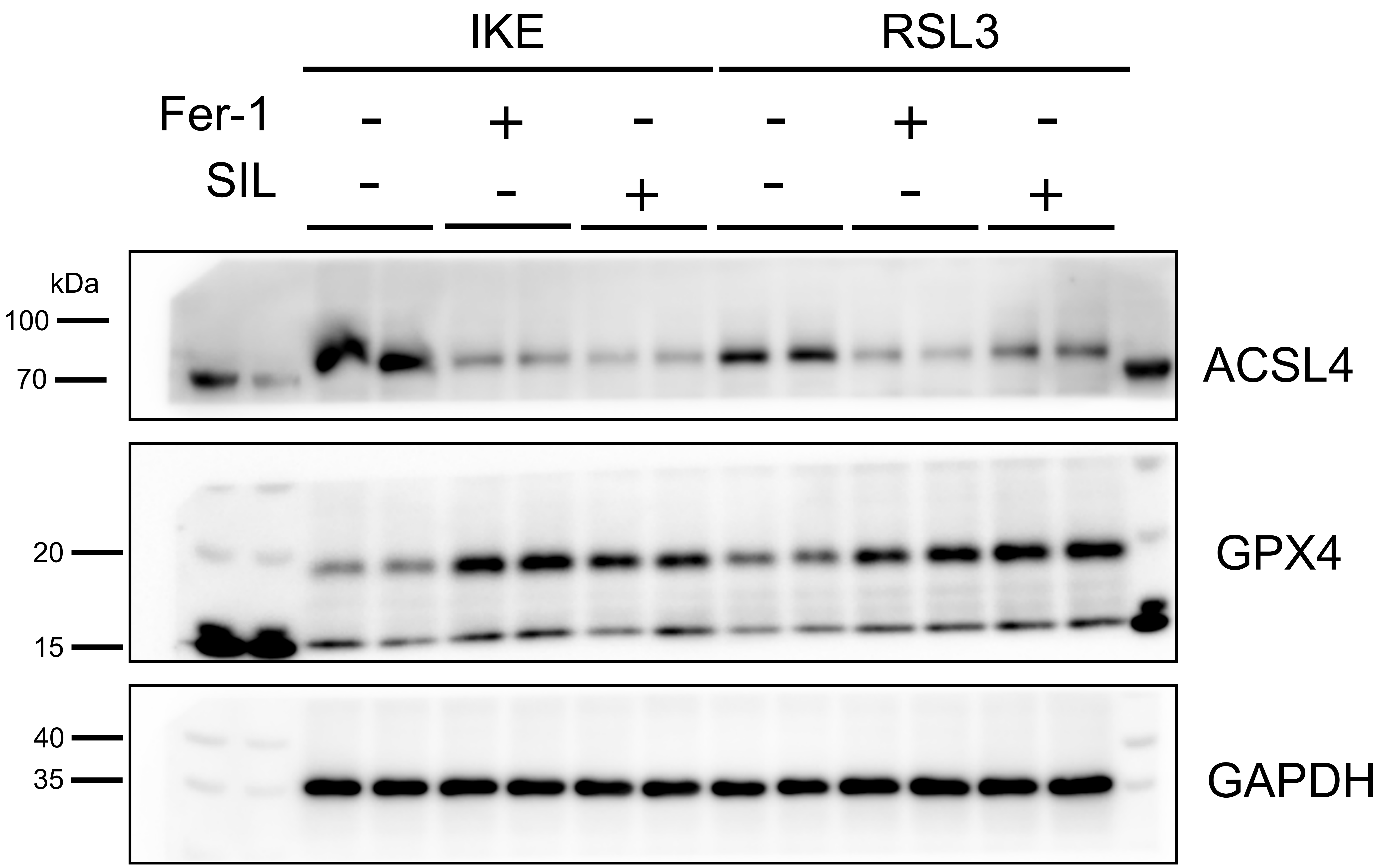

Supplement: Supplementary file 2 — Western Blot raw data [file 41419_2024_7309_MOESM2_ESM.pdf]
